# LIN-42, the *Caenorhabditis elegans* PERIOD homolog, Negatively Regulates MicroRNA Transcription

**DOI:** 10.1371/journal.pgen.1004486

**Published:** 2014-07-17

**Authors:** Roberto Perales, Dana M. King, Cristina Aguirre-Chen, Christopher M. Hammell

**Affiliations:** Cold Spring Harbor Laboratory, Cold Spring Harbor, New York, United States of America; Massachusetts General Hospital and Harvard Medical School, United States of America

## Abstract

During *C. elegans* development, microRNAs (miRNAs) function as molecular switches that define temporal gene expression and cell lineage patterns in a dosage-dependent manner. It is critical, therefore, that the expression of miRNAs be tightly regulated so that target mRNA expression is properly controlled. The molecular mechanisms that function to optimize or control miRNA levels during development are unknown. Here we find that mutations in *lin-42*, the *C. elegans* homolog of the circadian-related *period* gene, suppress multiple dosage-dependent miRNA phenotypes including those involved in developmental timing and neuronal cell fate determination. Analysis of mature miRNA levels in *lin-42* mutants indicates that *lin-42* functions to attenuate miRNA expression. Through the analysis of transcriptional reporters, we show that the upstream *cis-*acting regulatory regions of several miRNA genes are sufficient to promote highly dynamic transcription that is coupled to the molting cycles of post-embryonic development. Immunoprecipitation of LIN-42 complexes indicates that LIN-42 binds the putative *cis-*regulatory regions of both non-coding and protein-coding genes and likely plays a role in regulating their transcription. Consistent with this hypothesis, analysis of miRNA transcriptional reporters in *lin-42* mutants indicates that *lin-42* regulates miRNA transcription. Surprisingly, strong loss-of-function mutations in *lin-42* do not abolish the oscillatory expression patterns of *lin-4* and *let-7* transcription but lead to increased expression of these genes. We propose that *lin-42* functions to negatively regulate the transcriptional output of multiple miRNAs and mRNAs and therefore coordinates the expression levels of genes that dictate temporal cell fate with other regulatory programs that promote rhythmic gene expression.

## Author Summary

MicroRNAs play pervasive roles in controlling gene expression throughout animal development. Given that individual microRNAs are predicted to regulate hundreds of mRNAs and that most mRNA transcripts are microRNA targets, it is essential that the expression levels of microRNAs be tightly regulated. With the goal of unveiling factors that regulate the expression of microRNAs that control developmental timing, we identified *lin-42*, the *C. elegans* homolog of the human and *Drosophila period* gene implicated in circadian gene regulation, as a negative regulator of microRNA expression. By analyzing the transcriptional expression patterns of representative microRNAs, we found that the transcription of many microRNAs is normally highly dynamic and coupled aspects of post-embryonic growth and behavior. We suggest that *lin-42* functions to modulate the transcriptional output of temporally-regulated microRNAs and mRNAs in order to maintain optimal expression of these genes throughout development.

## Introduction

MicroRNAs (miRNAs) are non-coding RNA molecules that post-transcriptionally regulate gene expression [1]. The maturation of miRNAs is a stepwise process that begins with the RNA polymerase II-dependent transcription of long capped and polyadenylated primary miRNAs (pri-miRNAs) [2], [3]. Most pri-miRNAs are then endonucleolytically cleaved by the nuclear Microprocessor complex, composed of Drosha (an RNase III enzyme) and its binding partner Pasha, to yield a ∼70 nt precursor miRNA hairpin (pre-miRNA) [4]. After export to the cytoplasm, the pre-miRNA is cleaved by Dicer (a second Type III RNase) yielding a ∼22 nt duplex that consists of the mature miRNA and its corresponding passenger RNA [5], [6]. The mature single-stranded ∼22 nt miRNA is then loaded into the Argonaute and GW182 to form the miRNA-induced Silencing Complex (miRISC) [7]–[9]. Through partial complementary base-pairing between the miRNA and target mRNA, the miRISC complex negatively regulates gene expression by either translational repression or mRNA degradation [7], [10]. *In vivo*, target mRNA down-regulation is directly proportional to the amount of miRNA associated with miRISC [1].

Experimental and computational approaches indicate that an individual miRNA can bind to and regulate hundreds of mRNAs and that the majority of protein-coding genes are miRNA targets [11]–[14]. As such, miRNAs have been implicated in a variety of developmental and cellular processes including cell fate specification, proliferation and apoptosis [15]–[19]. In many of these scenarios, the expression of distinct miRNAs is tightly controlled and/or the individual steps of miRNA biogenesis are actively regulated at either the transcriptional or post-transcriptional level by sequence-specific transcription factors or RNA-binding proteins, respectively. For example, some regulatory proteins control miRNA biogenesis by directly binding structural elements within the pri- or pre-miRNA transcript whereas others broadly impact global miRNA biogenesis by inhibiting enzymes required for general miRNA processing and/or activity [20]. Importantly, many of the proteins that regulate miRNA biogenesis are highly conserved and mutations in these genes result in a variety of developmental disorders and diseases [20].

The *C. elegans* heterochronic pathway has been instrumental to our understanding of the principles of miRNA-mediated gene regulation and for the identification of components that are required to control miRNA expression, metabolism and activity [21]. Post-embryonic development in *C. elegans* proceeds through a series of four larval stages, punctuated by molts, in which the temporal and spatial patterns of cell division and differentiation are tightly orchestrated and invariant [22]. Heterochronic genes organize temporal patterns of development by controlling stage-specific gene expression. Defects in heterochronic genes cause animals to display temporal cell fate transformations including either the inappropriate skipping or reiteration of stage-specific patterns of cell divisions [23]. An overarching feature of the heterochronic pathway is that many protein-coding genes that are important for controlling temporal patterning are post-transcriptionally regulated by miRNAs [16], [24]–[28]. In this context, miRNAs are expressed at defined times during post-embryonic development and function as molecular switches to inhibit earlier patterns of development and promote the emergence of later gene expression profiles. Throughout post-embryonic development, the expression of heterochronic miRNAs is regulated at both the transcriptional and post-transcriptional levels [20], [29]–[32]. In addition, mutations that alter heterochronic miRNA expression often display strong temporal patterning and behavioral phenotypes [16], [33]–[36].

**Figure 1:**
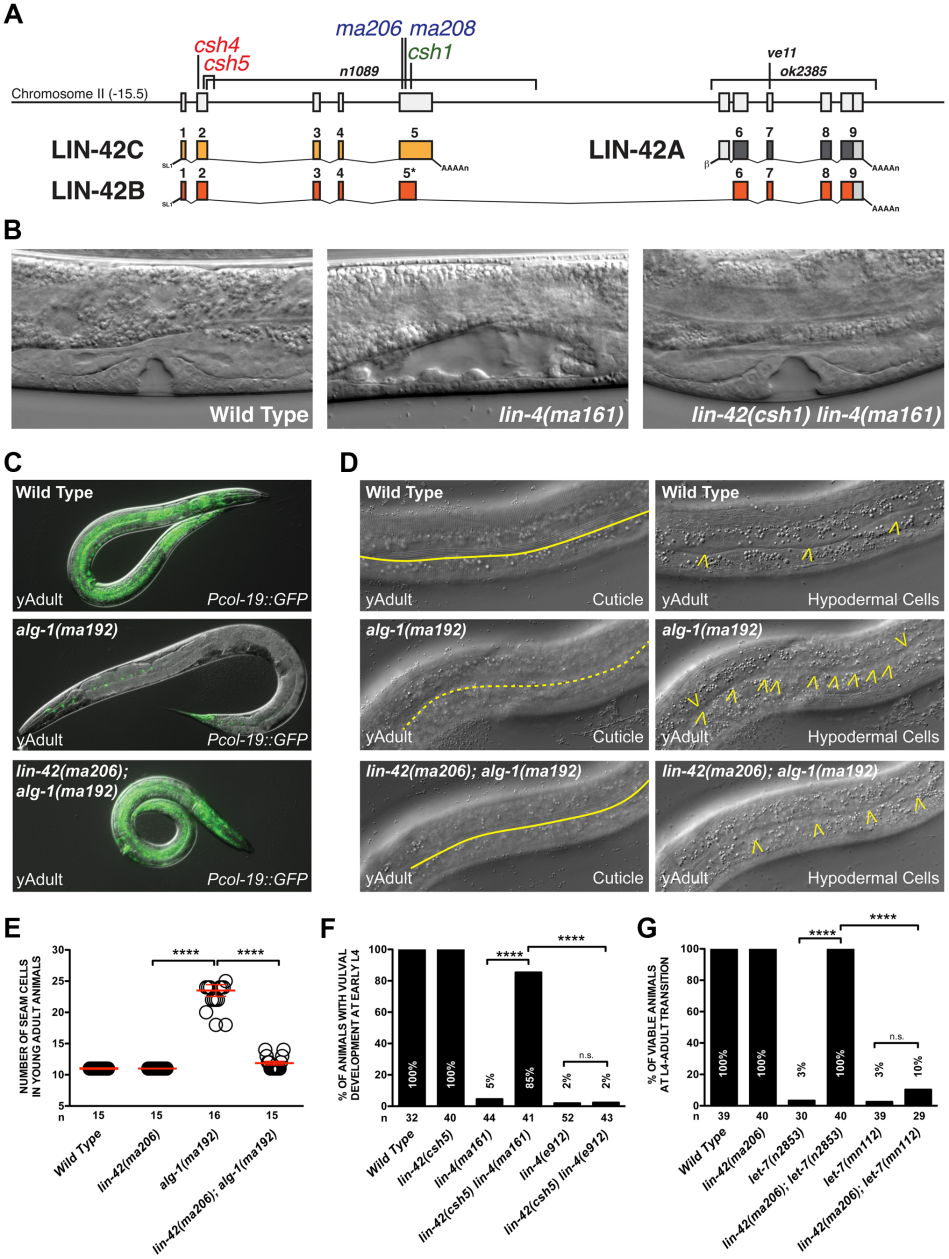
Mutations in *lin-42* suppress defects in temporal gene expression and cell lineage phenotypes of heterochronic miRNA mutants. (A) The genomic locus of *lin-42* and the corresponding location of the mutations identified in this screen. Red labeled alleles were identified in *lin-4(ma161)*, blue in *alg-1(ma192)* and green in *let-7(n2853)* genetic backgrounds. Alleles labeled in black have been previously described [39], [47], [48]. (B) Defects in vulval cell fate specification in *lin-4* mutant animals are corrected by *lin-42* mutations. (C) Adult-specific *Pcol-19::GFP* expression patterns in wild-type, *alg-1(ma192)* and *lin-42(ma206); alg-1(ma912)* animals. (D) Adult-specific alae phenotypes and seam cell phenotypes of wild-type, *alg-1(ma192)* and *lin-42(ma206); alg-1(ma192)* animals. Solid yellow lines indicate complete alae whereas dashed yellow lines indicate an absence of alae structures. Yellow arrowheads indicate lateral seam cells. (E) Quantification of seam cell numbers of young adult wild-type, *lin-42(ma206)*, *alg-1(ma192)* and *lin-42(ma206); alg-1(ma192)* animals. Red error bars indicate standard deviation from the mean (SD) (F and G) *lin-42* mutations suppress vulval cell lineage and lethality phenotypes of hypomorphic alleles of heterochronic miRNAs but not null mutations of these genes. For E, F and G, four asterisks (****) indicate a highly significant association (the two-tailed P value is less than 0.0001) between groups and/or outcomes as measured by Fisher's exact test and n.s. equals no statistical significance.

While the regulatory strategies that dictate patterns of cell fate specification have rapidly emerged through the identification of conserved heterochronic genes, we still lack a deep understanding of how the temporal expression of heterochronic genes are coordinated with aspects of growth and behavior. This coupling is especially important as many post-embryonic cell division and cell fate specification events are intimately tied to the molting cycle [37], [38]. Surprisingly, most of the known genes required for molting do not dramatically alter temporal cell fates and only a few heterochronic genes disrupt the reiterative process of molting [23], [38]–[45]. The molting phenotypes associated with heterochronic mutants usually result from inappropriate temporal cell fate transformations that lead to either a cessation (for precocious heterochronic mutants) or an inappropriate reiteration (for retarded heterochronic mutants) of molting [16], [23]–[28], [31], [33], [34], [42]–[45]. To date, only a single heterochronic gene, *lin-42*, is known to alter both temporal patterning of cell fate specification and the precise timing of recurrent developmental events [39]. *lin-42* is the *C. elegans* homolog of human and *Drosophila* PERIOD and was initially identified as a heterochronic mutant that precociously executes adult-specific patterns of development after the third larval molt [46]–[48]. The *lin-42* locus is complex and encodes three protein isoforms (LIN-42A, LIN-42B and LIN-42C) that are expressed from two distinct promoters (Figure 1A) [39], [46]–[48]. During post-embryonic development, *lin-42* mRNA levels fluctuate over the molting cycles and peak once during each larval stage [39], [46]–[48]. While its precocious developmental phenotypes are similar to other heterochronic mutants, the periodic expression pattern of LIN-42 distinguishes it from other monotonically expressed heterochronic proteins. Therefore, *lin-42* has been proposed to play a more iterative role in developmental timing. However, its relationship to and interplay with other heterochronic genes has been difficult to establish at the molecular level. In addition to altering temporal patterns of development, mutations that disrupt the expression of LIN-42A and LIN-42B isoforms display dramatic defects in behavior and molting [39]. Specifically, *lin-42a/b* mutants alter the normally synchronous molting patterns displayed by wild-type animals and these defects frequently result in lethality [39]. Given that LIN-42 is a nuclear protein, an attractive hypothesis is that LIN-42 coordinates gene expression programs that control the molting cycles with regulatory pathways that mediate stage-specific cell lineage programs [48]. However, this potential role for LIN-42 remains elusive because 1) the molecular nature of LIN-42 activity is yet to be defined and 2) LIN-42 downstream targets that mediate iterative (molting) and sequential (cell fate patterning) gene regulatory programs are unknown.

In this study, we employed multiple forward genetic screens that were collectively geared to identify negative regulators of miRNA expression. As a product of this approach, we identified mutations in *lin-42* that suppress multiple stage-specific lineage defects associated with heterochronic miRNAs. Analysis of miRNA expression in *lin-42* mutant animals suggests that LIN-42 broadly functions to negatively regulate miRNA expression is therefore is likely to act in a variety of pathways that require miRNAs for proper cell fate specification. Consistent with this hypothesis, we find that *lin-42* also plays a role in the miRNA-mediated specification of asymmetric gene expression patterns in gustatory neurons. Analysis of LIN-42 interactions with chromatin suggests that LIN-42 potentially regulates the transcription of both miRNAs and mRNAs. We demonstrate, through the use of transcriptional reporters, that *lin-42* mutations alter the transcription of *lin-4* and *let-7*. Surprisingly, mutations that remove LIN-42 isoforms containing the conserved PAS domains (required for circadian gene regulation by human and *Drosophila* PERIOD) do not uncouple miRNA expression from the molting cycle but, instead, dramatically alter the transcriptional output of miRNA genes. We conclude that a key molecular function of *lin-42* is to dynamically inhibit the transcription of post-embryonically expressed miRNAs and mRNAs to ensure the robustness of developmental gene expression.

## Results

### *lin-42* functions during post-embryonic development to ensure proper temporal cell fate specification mediated by miRNAs

The inherent dependency of the heterochronic pathway on precisely controlled miRNA activity provides a unique genetic context to identify components that control aspects of miRNA metabolism or expression. To accomplish this, we performed forward genetic screens in either *lin-4(ma161)*, *alg-1(ma192)* or *let-7(n2853)* mutant backgrounds to identify novel heterochronic mutations that correct the phenotypes associated with aberrant L1 to L2 (early), L2 to L3 (middle) or L4 to adult (late) cell fate transitions, respectively. These mutants are unique in that they express miRNAs at a much lower level than wild-type animals but do not completely eliminate their expression. *lin-4(ma161)* and *let-7(n2853)* mutations alter the conserved seed sequence of the mature miRNA and reduce levels of these miRNAs *in vivo* [16], [24]. Animals harboring *lin-4(ma161)* and *let-7(n2853)* mutations are phenotypically indistinguishable from null mutants and reiterate L1- and L4-specific cell fates, respectively (Table 1) [16], [24]. *alg-1(ma192)* mutations alter one of the two miRNA-specific Argonautes and disrupt the ability of processed miRNAs to repress downstream target mRNAs [49]. Animals harboring the *alg-1(ma192)* mutation inappropriately express *hbl-1* (the major miRNA target of miR-48, miR-241, and miR-84) in the L3 stage and reiterate L2-specific seam cell division patterns [49]. Consistent with the defects associated with the misregulation of each of these stage-specific transitions, *lin-4(ma161)*, *alg-1(ma192)* and *let-7(n2853)* animals display highly penetrant heterochronic phenotypes and fail to express adult-specific gene regulatory programs, including the expression of the adult-specific *Pcol-19::GFP* transcriptional reporter (Table 1). Suppressors of the retarded heterochronic phenotypes in each of these genetic backgrounds were identified as F2 progeny of mutagenized animals that were able to restore normal development (Figure 1C). Five mutants (*ma206*, *ma208*, *csh1*, *csh4 and csh5*) were able to suppress multiple retarded heterochronic phenotypes associated with all three mutant backgrounds. Each mutant mapped to a single locus on chromosome II (Figure 1A and Table 1), and subsequent SNP-SNP mapping and sequencing results demonstrated that all five alleles contain mutations that lie within *lin-42* and would be predicted to create a premature truncation of the *lin-42b* or *lin-42c* open reading frames (Figure 1A and Table S1) [50]. Consistent with previous analyses of *lin-42* mutations, animals harboring the *ma206*, *ma208*, *csh1*, *csh4* or *csh5* allele display highly-penetrant precocious heterochronic phenotypes (Table 1) which were rescued with a fosmid containing the genomic fragment of the wild-type *lin-42* gene [46]–[48]. Mutations in *lin-42* have been demonstrated to suppress heterochronic phenotypes associated with multiple heterochronic mutants, including *lin-4* and *let-7* [16], [46], [47], [51]. In these reports, only terminal cell lineage phenotypes, including a correction of the L4-to-adult vulval bursting phenotypes, restoration of adult-specific expression of *Pcol-19::GFP*, and formation of adult-specific alae were assayed.

**Table 1:** *lin-42* mutations suppress heterochronic phenotypes of multiple heterochronic miRNAs.

			Precocious alae		Adult alae		Adult	
#	Strain	Genotype^a^	formation at L4^b^	*n = *	formation^b^	*n = *	*col-19_pro_::GFP*	*n = *
1	VT1367	wild-type	0	30	100	30	100	37
2	HML252	*lin-42(n1089)*	80	30	100	31	100	40
3	VT2187	*lin-42(ma206)*	70	26	100	22	100	38
4	VT2198	*lin-42(ma208)*	38	48	100	41	100	27
5	HML18	*lin-42(csh1)*	47	30	100	24	100	42
6	HML43	*lin-42(csh4)*	40	23	100	20	100	30
7	HML44	*lin-42(csh5)*	60	34	100	25	100	30
8	VT2077	*lin-31(n1053)* c	0	30	100	30	100	34
9	VT2079	*lin-31(n1053); alg-1(ma192)* c	0	30	10	29	0	33
10	VT2383	*lin-42(ma206); alg-1(ma192)*	9	23	100	22	100	37
11	HML6	*lin-4(e912)*	0	30	0	19	0	24
12	HML7	*lin-42(ma206) lin-4(e912)*	0	30	48	23	100	24
13	HML264	*lin-42(csh5) lin-4(e912)*	0	30	30	30	100	26
14	HML2	*lin-4(ma161)*	0	30	0	29	0	23
15	HML3	*lin-42(ma206) lin-4(ma161)*	0	30	100	20	96	23
16	HML21	*lin-42(csh1) lin-4(ma161)*	19	27	37	35	100	35
17	HML24	*lin-42(csh4) lin-4(ma161)*	23	44	42	33	100	35
18	HML25	*lin-42(csh5) lin-4(ma161)*	9	43	54	28	100	35
19	CB1309	*lin-2(e1309)* d	0	20	100	20	-	-
20	HML9	*lin-2(e1309) unc-3(e151) let-7(mn112)* d	0	20	0	22	0	40
21	HML15	*lin-42(ma206); unc-3(e151) let-7(mn112)* d	0	21	33	30	-	-
22	HML20	*lin-42(csh1); unc-3(e151) let-7(mn112)* d	0	22	0	22	55	31
23	HML11	*lin-2(e1309) let-7(n2853)* d	0	30	27	26	17^e^	35
24	HML14	*lin-42(ma206); let-7(n2853)*	0	30	100	30	100	30

a Animals contain *maIs105*, an adult-specific *col-19pro::GFP* reporter that is integrated on chromosome V.

b Presence and quality of cuticular alae structures were assayed by Normarski DIC optics. Only one side of each animal was scored.

For most alleles of *lin-42*, L4-staged animals expressed adult alae but alae were not continuous (contained gaps) along the entire lateral.

c Animals harboring the *alg-1(ma192)* mutation display a 100% vulval bursting phenotype. The *lin-31(n1053)* mutation suppresses the vulval bursting phenotypes and causes *lin-31(n1053); alg-1(ma192)* animals to display a 100% vulvaless (Vul) phenotype. The *lin-31(n1053)* allele does not alter the seam cell heterochronic phenotypes of *alg-1(ma192)*.

d *lin-2(e1309)* mutations cause animals to lack a vulva but do not alter temporal patterning of the hypodermal seam cells. The *lin-2(e1309)* allele was included in strains that contain *let-7* mutations to prevent the highly penetrant vulval bursting phenotypes.

We next sought to determine whether the *lin-42* mutations we isolated suppressed only terminal heterochronic phenotypes or if they corrected additional stage-specific cell lineage defects associated with *lin-4(ma161)*, *let-7(n2853)* and *alg-1(ma192)* mutations. To test if our new *lin-42* mutants correct retarded cell lineage phenotypes, we compared multiple hypodermal cell lineages in *lin-4(ma161)*, *alg-1(ma192)*, and *let-7(n2853)* single mutants to double mutants that also harbored the individual *lin-42* candidate suppressor mutations. *lin-4* animals lack vulval structures as a consequence of reiterating L1-specific developmental programs in the hypodermis and failing to interpret inductive cues from the anchor cell that initiate vulval morphogenesis at the L3 stage [40], [52]. The vulvaless (Vul) phenotypes of *lin-4(ma161)* animals are highly penetrant (Figure 1B, F) and are almost completely suppressed by *lin-42*(*ma206*), *lin-42(ma208)*, *lin-42(csh1)*, *lin-42(csh4)* and *lin-42(csh5)* (Figure 1B, F). These results indicate that *lin-42* functions to control cell fate specification in at least the mid-L3 stage, when the vulval precursors are spatially patterned.

The ability of several of these suppressors to alleviate hypodermal cell lineage phenotypes in miRNA hypomorphic mutants was not limited to the vulval cell lineage. The lateral seam cells of *lin-4(ma161)*, *alg-1(ma192)*, and *let-7(n2853)* animals display altered temporal cell fate specification and also fail to terminally differentiate at the L4 molt. As a consequence, *lin-4(ma161)*, *alg-1(ma192)*, and *let-7(n2853)* animals lack alae structures as young adults (Table 1, Figure 1D). The alae phenotypes in *lin-4(ma161)*, *alg-1(ma192)*, and *let-7(n2853)* mutants was strongly suppressed by the *lin-42(ma206)* allele (Table 1). *alg-1(ma192)* mutants reiterate L2-specific seam cell division programs due to the inappropriate perdurance of *hbl-1* expression at the L3 stage [49]. As a consequence, young adult *alg-1(m192)* animals harbor supernumerary seam cells (23.5+/−3.78; WT = 11) (Figure 1D, E). *lin-42(ma206)* mutations strongly suppress the L2-to-L3 heterochronic phenotypes of *alg-1(ma192)* mutants as *lin-42(ma206); alg-1(ma192)* animals exhibit a significant reduction in the number of supernumerary seam cells (11.9+/−1.3) and display normal adult alae (Figure 1D and E). Therefore, *lin-42* has a role in controlling L2-to-L3 temporal cell fate transitions.

### *lin-42* mutations do not suppress lineage defects and lethal phenotypes associated with *lin-4(0)* and *let-7(0)* alleles

We asked whether our new *lin-42* alleles could suppress the heterochronic phenotypes associated with *lin-4(e912)* and *let-7(mn112)* null mutants to a level similar to that observed with the hypomorphic alleles used in our initial screens. To test this, we compared aspects of vulval cell proliferation and morphogenesis at the early L4 stage in *lin-4(ma161)*, *lin-42(csh5) lin-4(ma161)*, *lin-4(e912)* and *lin-42(csh5) lin-4(e912)* mutants to those of similarly staged wild-type and *lin-42(ma205)* animals. Lowering *lin-42* function in the context of the hypomorphic *lin-4(ma161)* background results in a strong restoration of vulval development with 85% of animals exhibiting induction/proliferation and invagination of P cells from the larval cuticle (Figure 1B and F). Surprisingly, 42% percent of *lin-42(csh5) lin-4(ma161)* animals exhibited morphologically normal adult vulva and were competent for egg laying (n = 100). In contrast, reducing *lin-42* activity in *lin-4(e912)* animals has little or no effect on P cell proliferation and vulval morphogenesis (Figure 1F). *lin-42* exhibits a similar genetic relationship to *let-7* mutations. Both hypomorphic (*n2853*) and null (*mn112*) alleles of *let-7* display highly penetrant vulval bursting phenotypes at the L4-to-adult transition (Figure 1G) [16], [28]. *lin-42* mutations almost completely suppress the lethality associated with larval-to-adult transitions in *let-7(n2853)* animals but do not statistically improve the viability of *let-7(mn112)* adults (Figure 1G). These results strongly suggest that *lin-42* mutations are not bypass suppressors of *lin-4* or *let-7* mutant phenotypes but likely require a minimum level of *lin-4* or *let-7* activity for suppression.

### *lin-42* loss-of-function mutations lead to an overproduction of many *C. elegans* miRNAs

**Figure 2:**
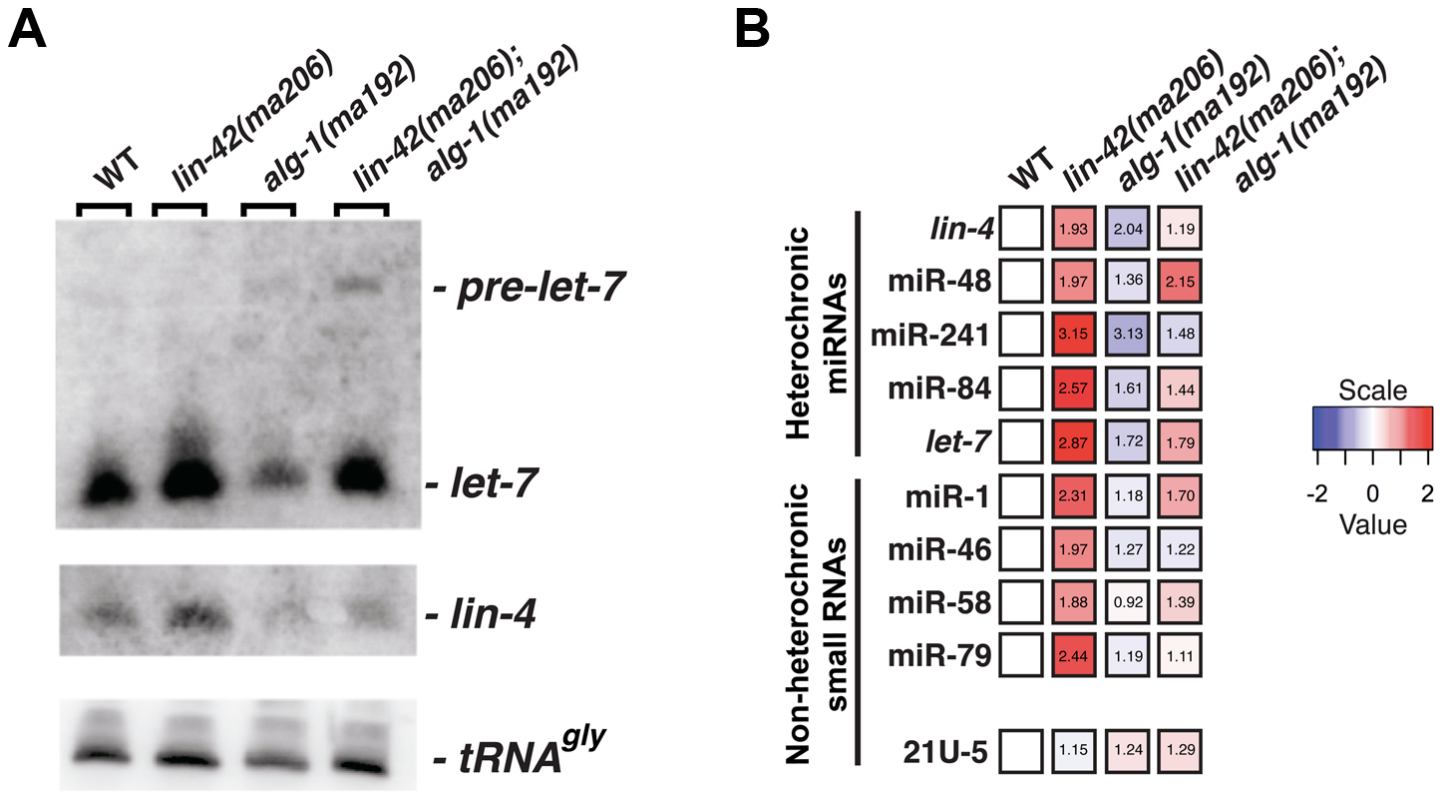
*lin-42* mutations lead to the overexpression of several miRNAs. (A) Small RNA northern analysis of 20 µg of total RNA extracted from morphologically staged, young adult wild-type, *lin-42(ma206)*, *alg-1(ma192)* and *lin-42(ma206); alg-1(ma192)* animals. Blots were probed sequentially for the indicated miRNAs. tRNAGly serves as a loading control. (B) The results of miR-TaqMan assays to quantify the levels of mature miRNAs in wild-type, *lin-42(ma206)*, *alg-1(ma192)* and *lin-42(ma206); alg-1(ma192)* animals. Notice that *lin-42(ma206)* displays the highest levels of miRNAs relative to the other genotype backgrounds. Data represent 3 biological replicates with 3 technical replicates each. Heat map colors are shown as log 2 scale as indicated and within each individual assay. Red indicates an increase in miRNA expression and blue indicates a reduction in mature miRNA levels. Numbers within each box indicate standard fold change when compared to wild-type samples.

One mechanism by which *lin-42* mutations could suppress multiple hypomorphic miRNA mutants would be that *lin-42* normally functions to repress some aspect of miRNA metabolism. To directly test this hypothesis, we measured the abundance of several mature miRNAs when *lin-42* function is compromised. Northern blot analysis of total RNA extracted from morphologically-staged, young adult animals demonstrates that the total amount of *lin-4* and *let-7* miRNAs in *alg-1(ma192)* mutants is 1–1.5 fold lower than the levels found in wild-type animals (Figure 2A and B). In addition to reducing the levels of mature *let-7* miRNA, *alg-1(ma192)* animals display a slight reduction in pre-miRNA processing and accumulate the pre-*let-7* hairpin precursor. This under-accumulation phenotype of mature *lin-4* and *let-7* miRNAs in *alg-1(ma192)* mutants is suppressed when *lin-42* function is compromised (Figure 2A). Consistent with our hypothesis that *lin-42* normally inhibits miRNA biogenesis, similarly-staged *lin-42(ma206)* mutants over-accumulate both *lin-4* and *let-7* miRNAs (Figure 2A). While the amount of mature *let-7* miRNA increases in *lin-42(ma206); alg-1(ma192)* double mutants, the ratio of *pre-let-7* to mature *let-7* miRNA is similar to that detected in *alg-1(ma192)* single mutants (Figure 2A). Therefore, although mature *let-7* miRNA over-accumulates in *lin-42(ma206)* mutants, there is no change in *pre-let-7* to mature *let-7* processing efficiency as compared to wild type. These data suggest that *lin-42* mutations alter aspects of miRNA expression upstream of pre-miRNA processing.

To determine if *lin-42* plays a more broad role in modulating miRNA expression, we employed real-time quantitative PCR to measure the expression levels of additional miRNAs in morphologically-staged, young adult wild-type, *lin-42(ma206)*, *alg-1(ma192)* and *lin-42(ma206); alg-1(ma192)* animals. We measured a variety of miRNAs that display tissue-specific and temporal expression patterns that are distinct from *lin-4* and *let-7* miRNAs [35], [53]–[58]. For comparison, we also assayed the expression of two additional small nuclear RNAs (U18 and sn2343) as well as two 21U RNAs that associate with PRG-1, a distinct Argonaute involved in the *C. elegans* piRNA pathway [59]–[61]. Consistent with the observation that *alg-1(ma192)* mutations broadly affect miRNA expression, the abundance of all miRNAs tested (*lin-4*, miR-48, miR-241, miR-84, *let-7*, miR-1, miR-46, miR-58 and miR-79) was decreased in *alg-1(ma192)* mutants (Figure 2B). The general miRNA under-accumulation phenotype displayed in *alg-1(ma192)* mutants was suppressed by removing *lin-42* function (Figure 2B). Importantly, the expression levels of the 21U-RNA transcripts were not significantly altered in *lin-42(ma206)* mutants (Figure 2B). Examination of miRNA expression in *lin-42(ma206)* mutants indicate that all tested miRNAs were overexpressed from ∼1.8 to ∼3.2 fold when compared to similarly-staged wild-type animals (Figure 2B). miRNA stability is dependent on a variety of factors, including the expression levels of the Argonaute components of miRISC [62]. To determine if the increase in miRNA levels in *lin-42* mutant backgrounds was due to the overexpression of the *C. elegans* miRNA-specific Argonautes (ALG-1 and ALG-2), we quantified the levels of functional ALG-1 and ALG-2 fluorescent reporters in animals with reduced *lin-42* activity. The results of this analysis, presented in Figure S1, indicate that ALG-1 and ALG-2 expression is not altered in *lin-42(RNAi)* animals. Collectively, these results indicate that *lin-42* functions to negatively regulate the expression of a wide range of miRNAs.

### *lin-42* suppresses dosage-dependent phenotypes of non-heterochronic miRNA mutants

**Figure 3:**
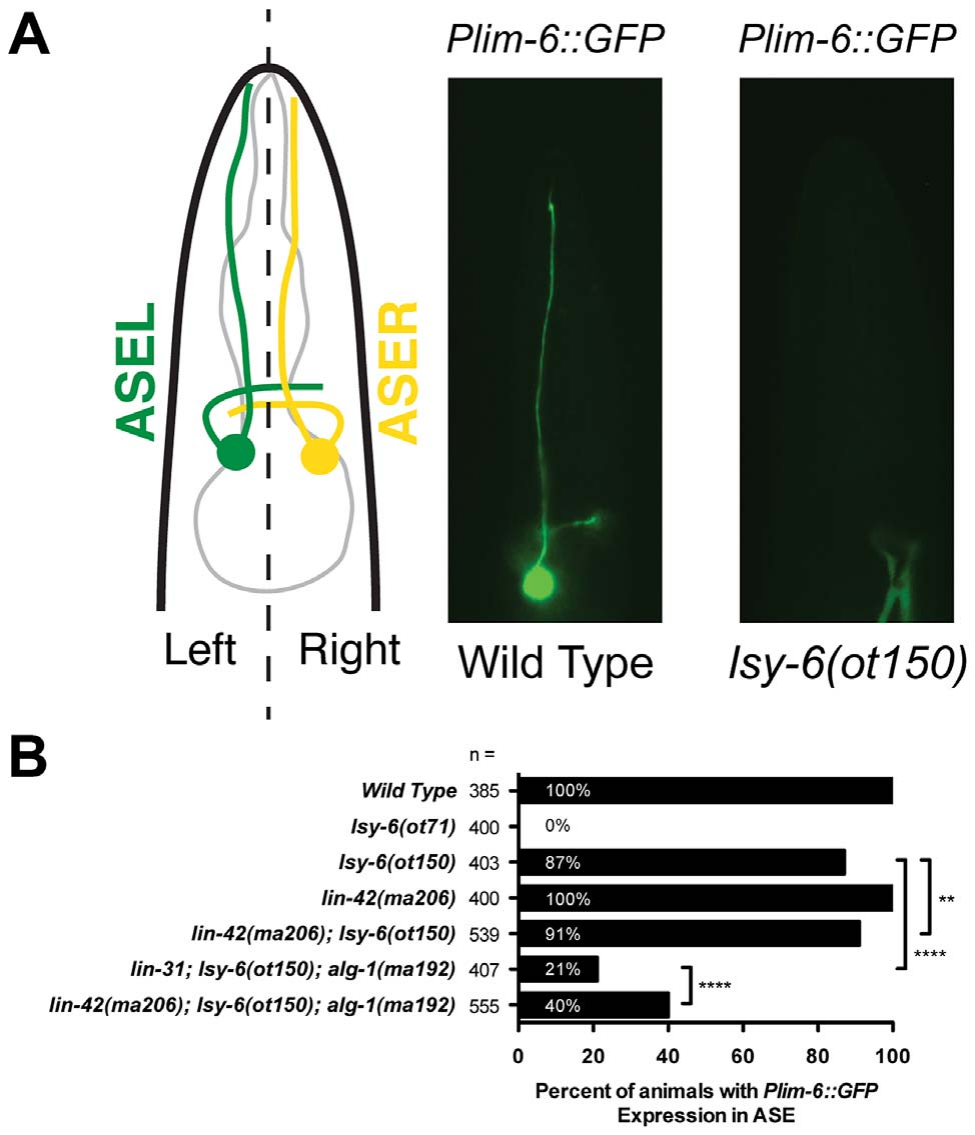
*lin-42* suppresses neuronal phenotypes associated with *lsy-6* miRNA-mediated cell fate specification. (A) A diagram of a *C. elegans* larva illustrating the location of the gustatory neurons, ASEL and ASER, whose asymmetric patterns of gene expression are controlled by the ASEL-specific expression of the *lsy-6* miRNA. Mutations in *lsy-6* result in animals that fail to express the ASEL-specific cell fate reporter *Plim-6::GFP*. (B) Quantification of *Plim-6::GFP* expression phenotypes of *lsy-6*, *lin-42*, *alg-1*, and *lin-42; alg-1* compound mutants. Four asterisks (****) indicate a highly significant association (the two-tailed P value is less than 0.0001) between groups and/or outcomes as measured by Fisher's exact test. Two asterisks (**) indicate a statistically significant association (P = 0.0242).

Because *lin-42* regulates the abundance of many miRNAs, we asked if *lin-42* functions in other gene regulatory pathways where controlling the expression levels of specific miRNAs is critical for proper cell fate determination. To test this idea, we examined how mutations in *lin-42* affected the cell fate specification of two bilaterally symmetric gustatory neurons, ASE left (ASEL) and ASE right (ASER). Normally, a complex gene regulatory network composed of miRNAs and transcription factors form a bi-stable, double-negative feedback loop that ensures mutually exclusive gene expression programs in ASEL and ASER neurons [63], [64]. A major determinant of the exclusive gene expression programs in these two neurons is the ASEL-specific expression of the *lsy-6* miRNA and the resulting down-regulation of its target, *cog-1*. Animals completely lacking *lsy-6* fail to down regulate COG-1 in ASEL, and, as a consequence, ASEL neurons in *lsy-6(ot71)* null mutants adopt an ASER cell fate [63]. These phenotypes can be monitored by a failure to express the *Plim-6::GFP* transcriptional reporter in ASEL in *lsy-6* mutants (Figure 3A). Importantly, *lsy-6*-mediated repression of *cog-1* is dosage-dependent; weak alleles of *lsy-6*, such as *ot150*, under-accumulate *lsy-6* miRNA as a consequence of reduced *lsy-6* transcription and result in a partially penetrant ASEL-to-ASER cell fate transformation phenotype (Fig. 3B) [64]. The *ot150* allele of *lsy-6* has been used in a variety of contexts as a sensitized genetic background to identify gene products that function in the miRNA pathway [65]–[67]. While 13% of animals harboring only the *lsy-6(ot150)* allele fail to maintain *Plim-6::GFP* in ASEL, the penetrance of this phenotype is partially suppressed in *lin-42(ma206); lsy-6(ot150)* double mutants (Figure 3B), suggesting that *lin-42* may play a modulatory role in neuronal cell fate specification. To further explore a potential role for *lin-42* in assuring proper neuronal cell fate specification, we developed a more sensitive assay for the *lsy-6*-mediated repression of *cog-1*. As previously mentioned, *alg-1(ma192)* mutants display defects in variety of miRNA-mediated processes, including developmental timing [49]. While the *alg-1(ma192)* mutation alone does not alter *Plim-6::GFP* expression in ASEL, combining *alg-1(ma192)* with *lsy-6(ot150)* results in a dramatic increase in ASEL to ASER cell fate mis-specification (Figure 3B). As with the suppression of *alg-1(ma192)* heterochronic phenotypes, reducing *lin-42* function significantly restores normal ASEL cell fate specification in *lsy-6(ot150); alg-1(ma192)* animals (Figure 3B). Because *lsy-6-*mediated cell fate specification is established during embryonic development, we conclude that *lin-42* functions throughout development and is critical for multiple miRNA-mediated developmental processes.

### miRNAs display dynamic expression patterns that are coupled to the molting cycles

**Figure 4:**
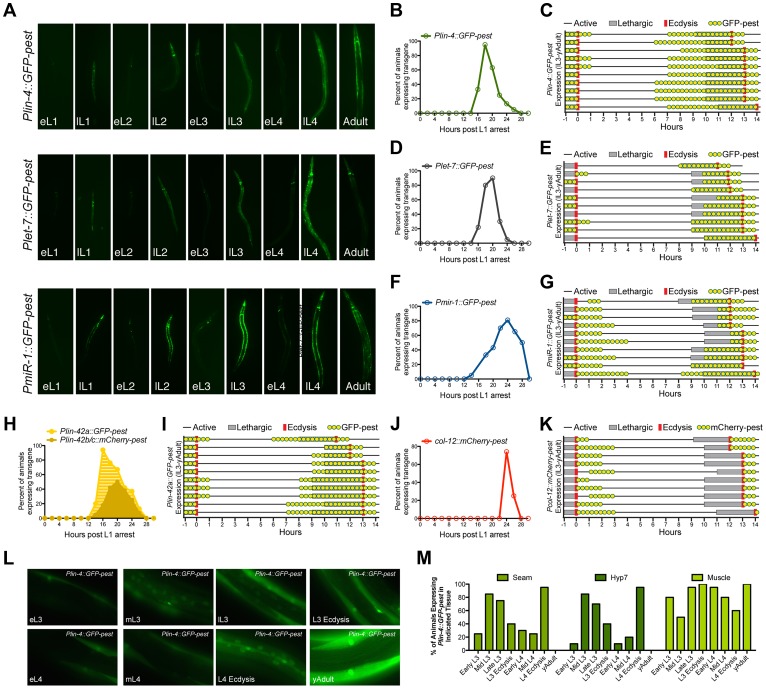
The promoters of three different miRNAs display dynamic expression patterns that are coupled to the larval molting cycle. (A) Transcriptional reporters for *lin-4*, *let-7* and *miR-1* drive GFP-pest expression in an oscillatory manner during *C. elegans* post-embryonic development. For each reporter, a single pulse of GFP expression is seen near the end of each inter-molt period of animals grown at 20°C (eL1, early L1 stage; lL1, late L1 stage). (B–K) GFP or mCherry expression profiles of animals expressing the indicated reporters. For panels B, D, F, H and J, larvae were synchronized by starvation-induced L1-diapause, fed and cultivated at 20°C. For panels C, E, G, I and H, a single animal in L3 lethargus (as judged by reduced movement and lack of pharyngeal pumping) was placed on an individual NGM plate seeded with OP50, grown at 20°C, and monitored for GFP or mCherry expression (yellow dots), the lethargus period (grey bars) or ecdysis (red bars). *Plin-4::GFP-pest* and *col-12::mCherry-pest* expression were monitored simultaneously in HML168, which co-expresses both reporters. (L) Representative fluorescent images of individual animals expressing the *Plin-4::GFP-pest* reporter at the indicated stage at 20°C. (M) Graphical representation of the percentage of animals expressing the *Plin-4::GFP-pest* reporter in any of the hyp7, seam or muscle cells at the indicated stage (n = 20 for each stage).

To characterize the spatial and temporal expression patterns of *lin-42*-regulated miRNAs, we generated a series of engineered transcriptional reporters that contain between 2 and 5 kB of genomic upstream regulatory sequence that drives the expression of GFP fused to an optimized proline-glutamate-serine-threonine-rich (PEST) sequence. PEST domains have been demonstrated, in a variety of heterologous systems, to accelerate the degradation of target proteins via the nuclear and cytoplasmic 26S proteasome [41], [68]–[71]. In contrast to transcriptional reporters that drive the expression of stable GFP, analysis of GFP-pest expression in *Plin-4::GFP-pest*, *Plet-7::GFP-pest* or *PmiR-1::GFP-pest* transgenic animals indicates that the expression of each transcriptional reporter is highly dynamic, with peak GFP-pest expression occurring once each larval stage (n>30 animals per time point)(Figure 4A) [29], [53], [55]. The highly dynamic nature of each expression pattern was then monitored in a population of worms that were transiently arrested at the L1 diapause and then developmentally synchronized by restoring bacterial food. For each of the *mir::GFP-pest* reporters, post-embryonic GFP-pest expression was first detected at approximately 14 hours (Figure 4B, D and F). Once transcriptionally activated, *Plin-4::GFP-pest* and *Plet-7::GFP-pest* reporters peak in expression by 18–20 hours and diminish with similar kinetics (Figure 4B and D). For animals expressing the *Plet-7::GFP-pest* reporter we monitored GFP-pest expression for longer periods after release from L1 arrest. Consistent with the highly pulsatile nature of this expression pattern, GFP-pest expression was reinitiated at 30 hours, which correlates with the later portions of the L2 stage (Figure S3). While transcriptional activation of the *Pmir-1::GFP-pest* reporter was also initiated at 14 hours post-L1 arrest, the peak of *Pmir-1::GFP-pest* expression occurred at a later time point, and diminished with slower kinetics, as compared to *Plin-4::GFP-pest* and *Plet-7::GFP-pest* expression (Figure 4F).

We then asked whether the temporal expression pattern of each *Pmir::GFP-pest* reporter was synchronized with defined stages of the molting cycle, specifically lethargus and ecdysis. To accomplish this, we isolated late-L3-staged transgenic animals and cultured them on separate nematode growth media (NGM) plates at 20°C. Individual animals were then monitored for GFP-pest expression in relation to the induction and termination of both lethargus and ecdysis (Figure 4C, E, and G). We find that the majority of animals which harbor the *Plin-4::GFP-pest* transgene cease GFP-pest expression by L3 ecdysis and resume expression by the mid-L4 stage. The pulse of *Plin-4::GFP-pest* expression at the L4 stage extends through the early portion of young adulthood and completely overlaps with the lethargus period in all animals (Figure 4C). *Plet-7::GFP-pest* expression followed a similar pattern (Figure 4E). However, GFP-pest expression was more variable at the L3-to-L4 transition and L4-specific induction of this transgene was primarily restricted to the lethargus period (Figure 4E). In contrast to the expression profiles of the *lin-4* and *let-7* reporters, induction of *Pmir-1::GFP-pest* expression began during, or immediately after, L3 ecdysis and persisted into the L4 stage. A second pulse of *Pmir-1::GFP-pest* expression completely overlapped with the L4 lethargus period and continued into early adulthood (Figure 4G). Collectively, these results suggest that the expression patterns of *lin-4*, *let-7* and *mir-1* are dynamic throughout development and that the cyclical transcription of these miRNAs is mediated by their cognate promoter sequences. Furthermore, these data show that, while each of the *Pmir::GFP-pest* reporters display pulsatile expression patterns, the transcriptional dynamics for each gene do not display a complete unity of phase in their expression profiles.

To compare the temporal expression patterns of these three miRNAs with that of *lin-42*, we constructed transgenic strains that expressed either *Plin-42a::GFP-pest* or *Plin-42b::mCherry-pest* and subjected these animals to the same time course analyses. It has been previously demonstrated that two independent promoters drive the expression of LIN-42A, LIN-42B and LIN-42C isoforms [39]. Consistent with these findings, *Plin-42a::GFP-pest* and *Plin-42b::mCherry-pest* reporters displayed highly pulsatile expression during the L1 stage with initiation and termination of expression at 12 and 28 hours post L1 arrest, respectively (Figure 4H). In addition, we find that *Plin-42a::GFP-pest* expression peaks at 16 hrs, immediately preceding the expression of the *Pmir::GFP-pest* reporters, while the peak of *Plin-42b::mCherry-pest* expression occurs at 20 hrs (Figure 4H). Detailed analysis of individual L3-to-adult animals indicates that *Plin-42a::GFP-pest* expression displays a temporal expression pattern that is highly similar to *Plin-4::GFP-pest* and *Plet-7::GFP-pest* expression (Figure 4I). Specifically, in all three reporters, *GFP-pest* expression diminishes prior to L3 ecdysis, resumes prior to the L4 lethargus period, and terminates immediately after L4 ecdysis (Figure 4I). In striking contrast to our *mir* and *lin-42* transcriptional reporters, *Pcol-12::mCherry-pest* expression does not occur during the molting cycle, but rather is exclusively expressed after each ecdysis (Figures 4J and 4K).

Previous analysis of *lin-4* and *let-7* expression indicates that these miRNAs are expressed in a variety of tissues, including the hypodermis, intestine and muscle [29], [35], [53], [57]. To determine if *Plin-4::GFP-pest* displays differential temporal expression patterns in a subset of these tissues, we conducted a detailed examination of GFP-pest expression from the early-L3 to the young adult stage. Twenty animals from each of eight morphologically-defined stages were imaged (Figure 4L and Figure S4) and then qualitatively scored for GFP-pest expression in seam cells, hyp7 cells or lateral muscle cells (Figure 4M). Expression of the *Plin-4::GFP-pest* reporter peaked in hyp7 and seam cells at the mid- and late-L3 stage and then again at the late-L4 stage. In addition, the majority of animals exhibited a cessation of hyp7 and seam cell *Plin-4::GFP-pest* expression immediately after L4 ecdysis (Figure 4M). In contrast, *Plin-4::GFP-pest* expression in muscle cells displayed a different transcriptional profile. In the majority of animals, expression of GFP-pest in muscles peaked at L3 ecdysis, gradually diminished throughout the remainder of the L4 stage, and increased again at the young adult stage (Figure 4M). These results suggest that, while *lin-4* is dynamically expressed once each larval stage, its promoter activity may be differentially regulated in distinct tissues.

### LIN-42 is enriched at promoters of coding and non-coding genes

**Figure 5:**
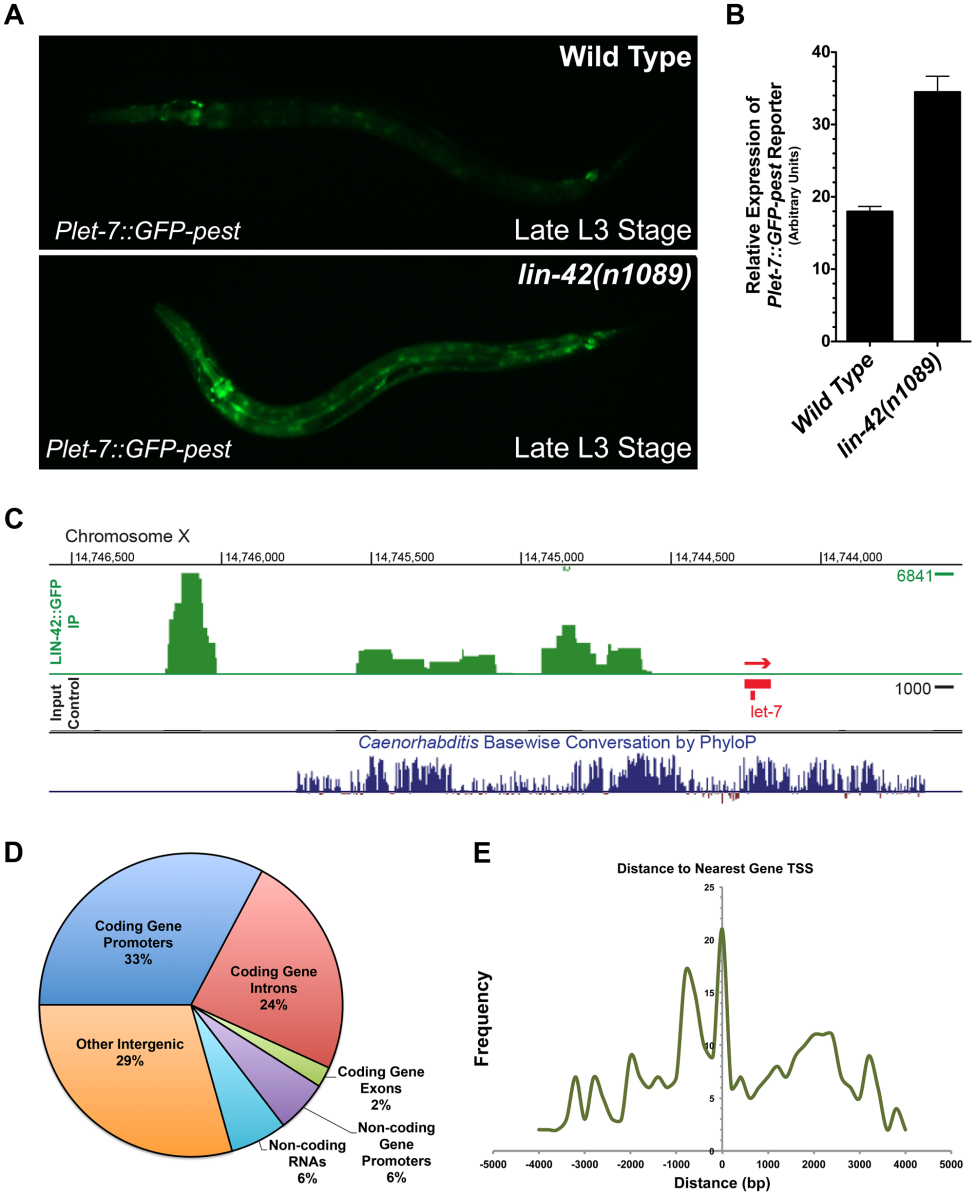
LIN-42 binds to the putative regulatory regions of both miRNAs and mRNAs. (A) *Plet-7::GFP-pest* expression is elevated in late L3-staged *lin-42(n1089)* animals compared to the expression in similarly-staged wild-type animals. (B) Quantification of whole-animal GFP expression in late L3-staged wild-type (n = 20) and *lin-42(n1089)* animals (n = 20). Error bars indicate the standard deviation from the mean (SD). (C) A representative image of the LIN-42::GFP binding sites within the *let-7* genomic region from ChIP-Seq experiments. (D) A pie chart indicating the distribution of the 413 high confidence LIN-42::GFP peaks that were assigned to a RefSeq list of gene models for *C. elegans* (ce10). (E) LIN-42::GFP binding sites are enriched upstream of the putative transcriptional start sites of both coding and non-coding regions of the genome.

Analysis of the *Plet-7::GFP-pest* and *Plin-4::GFP-pest* reporters in *lin-42* loss-of-function (lf) animals demonstrated that mutants that alter either *lin-42 b/c (lin-42(n0189))* or *lin-42a/b (lin-42(ok2385))* isoforms display elevated *Pmir-GFP-pest* expression in late larval development (Figure 5A, B and Figure S2). These altered temporal expression patterns suggested that *lin-42* may normally function to modulate aspects of miRNA transcription. To investigate the potential interactions between LIN-42 and transcriptional regulatory elements, we performed chromatin immunoprecipitation coupled to high throughput sequencing (ChIP-seq) using extracts prepared from animals harboring a functional, GFP-tagged allele of *lin-42* (Figure 5C). From two independent biological ChIP-seq replicates derived from separate L4-staged extracts, we obtained 413 high confidence peaks corresponding to chromosomal regions in which LIN-42 is enriched (see Table S2 and Materials and Methods). In agreement with the hypothesis that LIN-42 regulates *let-7* transcriptional activity, we find LIN-42 binding sites at conserved *let-7* promoter regions that have been previously demonstrated to control *let-7* expression (Figure 5C) [31], [35], [57]. Annotation of additional high confidence peaks revealed that 38% (158/413) of LIN-42 peaks fell within the promoters (defined as 2 kb upstream of each gene) of either coding or non-coding genes, 24% (99/413) fell within the introns of coding genes, 8% (34/413) fell within gene bodies and 29% (121/413) fell within other intergenic regions (Figure 5D). Comparison between LIN-42 peak frequency and their distribution relative to the closest annotated transcription start site (TSS) revealed that LIN-42 has two major regions of enrichment: 1) directly at TSSs and 2) at approximately 750 bp upstream of a TSS (Figure 5E). Of these high confidence peaks, 323 were also detected in LIN-42 ChIP-seq samples obtained using an antibody against endogenous LIN-42, suggesting that this list forms a short, but high confidence, group of LIN-42 target genes. A list describing all high-confidence annotated LIN-42 peaks is provided in Table S2. Using the Generic Gene Ontology Term Mapper, we found that numerous genes with high-confidence LIN-42 peaks can be categorized into groups that function in many diverse biological processes, including development, transport, small molecule metabolism, embryogenesis and growth (Table S3). Collectively, these results strongly suggest that LIN-42 plays a role (either directly or indirectly) in a broad range of biological processes and that it predominately interacts with the promoter regions of coding and non-coding genes to regulate their expression.

### *lin-42* negatively regulates the transcriptional output of *lin-4* and *let-7*

**Figure 6:**
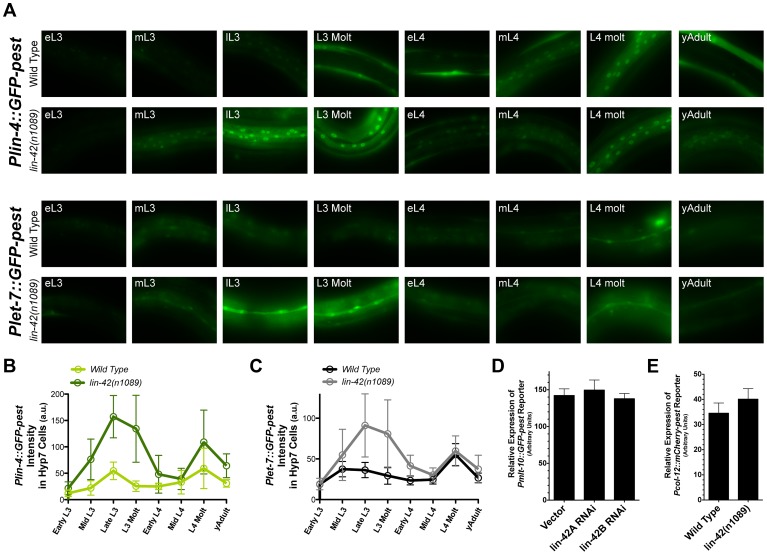
*lin-42* controls the output of *lin-4* and *let-7* transcription. (A) Representative expression patterns of the *Plin-4::GFP-pest* and *Plet-7::GFP-pest* transcriptional reporters in morphologically-staged wild-type and *lin-42(n1089)* animals grown at 20°C (early L3 to young adult). See Figure S4 for details. (B and C) A quantitative representation of gene expression profiles measured at the cellular level for both transcriptional reporters. GFP intensities for *Plin-4::GFP-pest* and *Plet-7::GFP-pest* were measured in the nuclei of hypodermal cells and seam cells, respectively. For *Plin-4::GFP-pest*, each data point in the graph represents the average from 200 nuclei. For *Plet-7::GFP-pest*, each data point represents the average from 50 seam cells. Error bars indicate the standard deviation from the mean (SD). (D) Quantitation of *Pmlt-10::GFP-pest* reporter expression in the hyp7 cells of lL4-staged F1 animals that have been exposed to bacteria expressing control RNAi (pDP129.36) or bacteria expressing dsRNAs against two isoforms of the *lin-42* gene (n = 20 for each experiment). Error bars indicate the standard error of the mean (SEM). (E) Quantitation of *Pcol-12::mCherry-pest* reporter expression in seam cells of young adult, wild-type (n = 20) or *lin-42(n1089)* animals (n = 20). Error bars indicate the standard error of the mean (SEM).

The genetic and regulatory relationships between *lin-42* and *lin-4* or *let-7*, as well as the overlapping temporal expression patterns of these three genes, suggest that *lin-42* may play a role in modulating the dynamics of *lin-4* and *let-7* transcriptional activity. To directly test the idea that *lin-42* regulates miRNA levels at the transcriptional level, we quantified the transcriptional profiles of *Plin-4::GFP-pest* and *Plet-7::GFP-pest* reporters in wild-type animals and *lin-42(n1089)* mutants. The *n1089* allele of *lin-42* deletes genomic sequences that eliminate the coding potential of the *lin-42b* and *c* isoforms (Figure 1A) and displays strong heterochronic phenotypes [39], [46], [47]. Importantly, these isoforms contain the domains, PAS-A and PAS-B, that most closely link LIN-42 to PERIOD, a protein involved in controlling the cyclical expression patterns of circadian-regulated genes [39], [47], [48], [72], [73]. We focused on quantifying the GFP intensities of 1) the hypodermal cells in L3-to-adult-staged *Plin-4::GFP-pest* animals and 2) the seam cells of similarly-staged *Plet-7::GFP-pest* animals. These tissues and stages were selected for analysis because the majority of well-characterized heterochronic phenotypes are detected in these tissues [16], [24], [39], [42], [46]–[48]. Expression levels for each transcriptional reporter were analyzed throughout eight defined and sequential stages that spanned from early L3 to young adult (Figure 6A–C and Figure S4). In agreement with our previous observations, expression of *Plin-4::GFP-pest* in hypodermal cells of wild-type animals is dynamic throughout development and displays two main peaks of GFP expression: one at the late-L3 stage and the other at the L4 molt (Figure 6A, B). Similar results are also observed in the seam cells of wild-type animals expressing the *Plet-7::GFP-pest* reporter (Figure 6B, C). One exception, however, is that that the first peak of *Plet-7::GFP-pest* expression occurs at the mid-L3 stage (Figure 6B, C). Surprisingly, we find that the cyclical pattern of expression of these reporters is not affected in animals carrying the *lin-42(n1089)* mutation; both *lin-42(n1089)* and wild-type animals display nearly identical *Plin-4::GFP-pest* and *Plet-7::GFP-pest* temporal expression patterns (Figure 6A, B and C). In contrast, the abundance of GFP-pest expression for each reporter is universally higher in *lin-42(n1089)* mutants as compared to similarly-staged wild-type animals (Figure 6 A, B and C). In the case of the *Plin-4::GFP-pest* reporter, higher levels of GFP-pest intensity are observed in hypodermal cells throughout all developmental stages, with the greatest difference occurring between the late-L3 and L3-molt stages (3.1 and 4.3 fold respectively)(Figure 6B). Interestingly, although *Plet-7::GFP-pest* expression in *lin-42(n1089)* mutants is also greater in seam cells between the late-L3 and L3-molt stages (2 fold each), *Plet-7::GFP-pest* expression in *lin-42(n1089)* and wild-type animals is practically indistinguishable from wild-type during the mid-L4 to the young adult stages (Figure 6C). Taken together, these results suggest that mutations that abolish the expression of PAS domain-containing LIN-42 isoforms do not alter the cyclical expression patterns of miRNA genes during development. Rather, these mutations alter the transcriptional output of miRNAs that display oscillatory expression patterns.

As demonstrated in Figure 5D and Table S2, LIN-42 binds the putative regulatory regions of multiple protein coding genes. This observation raises the possibility that LIN-42 may modulate the transcriptional output of other developmentally regulated genes, including those whose expression, like that of *lin-4* and *let-7*, is also linked to the molting cycle. To determine if *lin-42* mutants alter the transcriptional output of other cyclically expressed mRNAs, we observed the expression of two transcriptional reporters for genes involved in the molting process, *Pmlt-10::GFP-pest* and *Pcol-12::mCherry-pest*. In wild-type animals, *Pmlt-10::GFP-pest* transcription begins at the end of each larval period when the new cuticle is being synthesized [39], [41]. We monitored the expression of *Pmlt-10::GFP-pest* in F1 animals that had been exposed to control RNAi or two RNAi constructs that target all major isoforms of *lin-42* and induce precocious expression of *Pcol-19::GFP* and adult alae [74]. As with the expression of *Plin-4::GFP-pest* and *Plet-7::GFP-pest* reporters, the *Pmlt-10::GFP-pest* reporter maintained its normal, oscillatory pattern of expression in *lin-42(RNAi)* animals. Quantification of *Pmlt-10::GFP-pest* reporter expression at the late-L4 stage (where *Pmlt-10::GFP-pest* normally peaks [39], [41]) indicates that *lin-42* depletion does not alter the transcriptional output of the *mlt-10* promoter (Figure 6D). In addition, quantification of the *Pcol-12::mCherry-pest* reporter in young adult *lin-42(n1089)* animals also indicates that mutations in *lin-42* do not alter the temporal expression patterns or levels of the *col-12* promoter (Figure 6E). Therefore, while *lin-42* mutations alter the transcriptional output of the *lin-4* and *let-7* genes, *lin-42* does not play an essential role in controlling the oscillatory expression patterns or transcriptional output of all genes whose expression is tied to the molting cycle.

## Discussion

Using an unbiased genetic approach, we sought to identify factors that modulate the expression of miRNAs that are critical for controlling temporal patterns of development throughout post-embryonic development. Our strategy was two-fold: 1) we sought to identify suppressors of heterochronic miRNA mutant phenotypes characterized by stage-specific alterations in temporal patterning and 2) we focused on identifying suppressors that preferentially alleviate phenotypes that result from a reduction in, rather than a complete loss of, miRNA expression. These efforts identified *lin-42*, the *C. elegans* homolog of the circadian *period* gene, as a component that not only modulates heterochronic miRNA expression, but also regulates the expression of a wide range of broadly expressed, and functionally distinct, *C. elegans* miRNAs. Previous genetic analyses implicated *lin-42* as a heterochronic gene that normally inhibits the precocious expression of adult characteristics [39], [46]–[48]. The precise placement of *lin-42* in the developmental timing pathway has been difficult to incorporate due to the observation that *lin-42* mutations alter cell lineage programs that occur exclusively in late development, namely the transition from the L3 to the L4 stage [46]–[48], [51]. In addition, epistasis experiments with other developmental timing mutants suggest that its interaction with other heterochronic genes is complex [46], [47], [51], [75], [76]. Furthermore, unlike other components that control discrete aspects of temporal patterning and display monotonic expression patterns, *lin*-42 expression is highly dynamic, suggesting a reiterative role for it in the heterochronic pathway.

Results from our screens have identified five new alleles of *lin-42* that suppress the adult-specific gene expression defects of hypomorphic alleles of heterochronic miRNAs. We also find that *lin-42* corrects stage-specific cell fate specification defects present throughout larval and adult development in these miRNA mutants. These results indicate that *lin-42* functions iteratively to control temporal cell fate specification by controlling the transcription of distinct miRNAs. In addition, we demonstrate that our newly-identified *lin-42(lf)* mutants precociously express adult-specific programs and that these defects are suppressed by mutations in components of the miRNA machinery. Accordingly, these data suggest that *lin-42(lf)* heterochronic phenotypes are due to an overexpression of specific miRNAs that control temporal patterning. Also, our results demonstrate that *lin-42* mutations are not bypass suppressors of the heterochronic phenotypes displayed by *lin-4* and *let-7* null mutants, suggesting that *lin-42* suppresses retarded heterochronic phenotypes by increasing the expression of heterochronic miRNAs or enhancing their effectiveness in regulating miRNA targets.

Multiple lines of evidence described in this manuscript support the conclusion that LIN-42 regulates the transcription of a wide array of miRNAs. First, we investigated how our *lin-42* suppressor alleles affected the overall levels of a subset of miRNAs involved in developmental timing. *alg-1(ma192)* animals display profound defects in temporal cell fate specification and also under-accumulate both *lin-4* and *let-7* miRNAs. Genetic and molecular experiments indicate that *lin-42* suppresses *alg-1(ma192)-*dependent phenotypes by increasing the available amount of mature miRNAs. Second, *lin-42(ma206)* mutants over-accumulate multiple miRNAs, including those with no apparent role in developmental timing. Consistent with the hypothesis that *lin-42* functions in additional gene regulatory pathways that require miRNA activity, we demonstrated that *lin-42(lf)* mutants suppress phenotypes associated with the under-accumulation of a miRNA that is essential for proper neuronal cell fate specification. Because *lsy-6*-mediated regulation of *cog-1* expression is dosage-dependent, we speculate that *lin-42(lf)* mutations suppress neuronal cell fate specification defects by de-repressing *lsy-6* transcription in ASEL neurons.

In order to understand how *lin-42* may modulate miRNA expression, we pursued two lines of inquiry. First, we constructed a series of reporters that allowed us to measure, in detail, the transcriptional dynamics of multiple miRNAs in developing animals. Using these reporters, we found that several heterochronic miRNAs, such as *lin-4* and *let-7*, exhibit highly dynamic expression patterns that are synchronized with the expression of genes required for each molting cycle. Importantly, the expression of the *Plin-4::GFP-pest* and *Plet-7::GFP-pest* reporters coincided with the transcriptional activation of *lin-42*. Further analysis of the *lin-4* and *let-7* reporters in a *lin-42* mutant background indicated that one function of *lin-42* is to negatively regulate the transcriptional output of miRNA promoters. Therefore, LIN-42 functions in a manner similar to the human and *Drosophila* PERIOD proteins, which inhibit the transcription of circadian regulated genes [77], [78]. Second, it has previously been shown that LIN-42 is a nuclear protein, which suggests that it may play a role in directly regulating the pulsatile expression patterns of its downstream targets [48]. In order to explore potential roles for LIN-42 in directly controlling aspects of miRNA transcription, we performed ChIP-seq experiments to determine if LIN-42 interacts with the putative regulatory regions thought to control the expression of miRNAs and mRNAs. These experiments demonstrated that LIN-42 interacts with the promoters of non-coding genes (including *let-7*) as well as protein-coding genes, suggesting that *lin-42* may regulate the temporal expression of broad class of genes.

**Figure 7:**
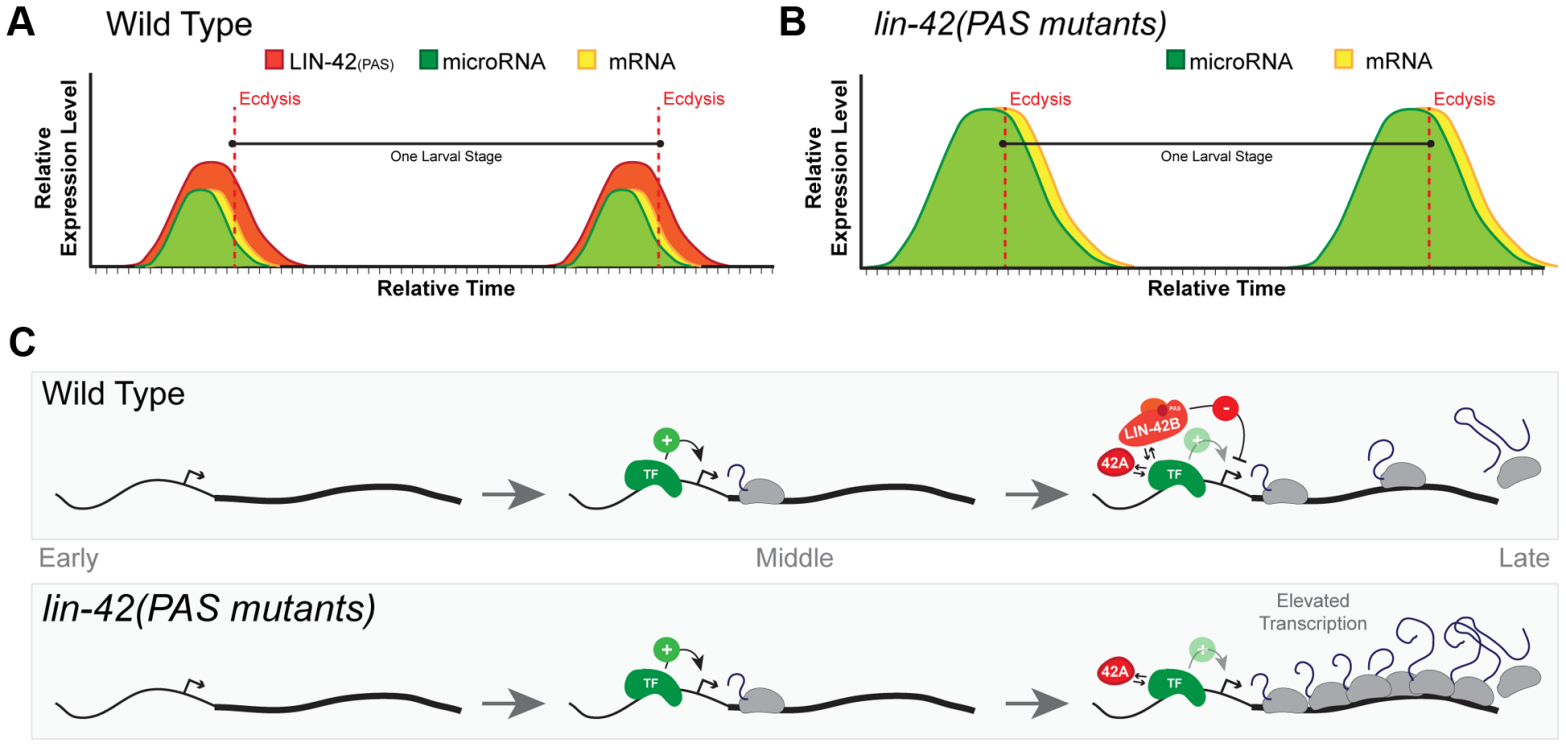
A model for *lin-42* function in regulating post-embryonic miRNA expression. (A) In wild-type animals, transcriptional activation of miRNAs and *lin-42*-regulated mRNAs is pulsatile and displays a peak of expression once each larval stage. The temporal expression of these miRNAs and mRNAs begins in the later portions of each stage and are coincident with behavioral and morphological events that demarcate the end of each larval stage. Several temporally-regulated miRNAs and mRNAs share similar patterns of expression that are coupled to the molting cycle. By ecdysis and initiation of a new larval stage, periodic expression of miRNAs, mRNAs and *lin-42* ceases. (B) The oscillatory expression patterns of miRNAs and mRNAs are maintained in animals that lack expression of LIN-42 isoforms containing the PAS domains. Only the transcriptional output of *lin-42*-regulated genes is altered, leading to the precocious phenotypes observed in these *lin-42* mutants. (C) Analyses of LIN-42 ChIP data and the expression patterns of the *lin-4* and *let-7* transcriptional reporters in *lin-42* mutants indicate that LIN-42 negatively regulates the transcriptional output of miRNA genes. We hypothesize that LIN-42 normally counteracts the transcriptional activity of one or more sequence-specific transcription factors (TF) that normally promote temporal expression. We would also predict that LIN-42 alters multiple aspects of transcription through its direct interaction with the TF or by binding to other cis-regulatory elements or to components of the transcriptional machinery. Mutations that alter only PAS domain-containing isoforms of LIN-42 fail to dampen transcriptional output. Importantly, this class of LIN-42 mutants would leave LIN-42A expression intact. Expression of LIN-42A would ensure normal periodic transcription of target genes and normal molting cycles and behaviors.

Given the role of human and *Drosophila period* in regulating circadian gene expression, we were surprised to find that animals harboring the *lin-42(n1089)* allele, which abolishes the expression of PAS-containing *lin-42* isoforms, maintained *lin-4* and *let-7* periodic expression patterns in later larval development. The PAS domains of human and *Drosophila* PERIOD are absolutely required to maintain the oscillatory expression patterns of circadian-regulated genes [72], [77], [78]. In our experiments, peak expression of the *lin-4* and *let-7* transcriptional reporters occurred at roughly the same developmental stages in both wild-type and *lin-42(n1089)* animals. Interestingly, although the temporal expression patterns were similar, the levels of each reporter were elevated (as high as four fold) in *lin-42(n1089)* mutants as compared to wild-type animals (Figure 7A and B). Notably, *lin-42(n1089)* mutations do not alter the expression of the *lin-42a* isoform, which has been implicated in controlling the periodicity of the molting cycle [39]. While the dissection of *lin-42* function will require further study, these findings are consistent with the modular nature of LIN-42 activities and suggest a novel role for the PAS domains of LIN-42 in regulating the transcriptional output of periodically expressed genes.

Based on our current observations, we propose a model in which each of the *lin-42* isoform functions to sculpt the dynamic transcription of both miRNAs and mRNAs. In out model, cis-regulatory elements within the promoters of specific miRNAs and mRNAs would be sufficient to drive periodic transcription. Regulatory elements within these sequences would be bound by a sequence-specific transcription factor (TF) that would promote the periodic transcription of these genes near the end of each larval stage (Figure 7C). Based on the role of PERIOD in other organisms and our data demonstrating that LIN-42 binds to the putative cis-regulatory elements of several miRNAs and mRNAs, we propose a model in which distinct isoforms of LIN-42 function to regulate the activity of the TF at multiple, genetically separable levels. Our evidence suggests that mutations that specifically disrupt isoforms containing the PAS domain (LIN-42B and LIN-42), fail to properly limit the transcriptional output of genes regulated by the temporal specific TF (Figure 7C). As a consequence, although these mutants display essentially normal temporal patterns of miRNA transcription, the elevated levels of heterochronic miRNAs lead to precocious developmental phenotypes. Importantly, mutations that only alter PAS-domain containing isoforms of LIN-42 retain the expression of LIN-42A (Figure 7B) [39]. Our model would also predict that mutations that disrupt LIN-42 isoforms that contain the conserved SYQ/LT domains (LIN-42A and LIN-42B) would have complex phenotypes with regard to periodic transcription. Indeed, animals harboring the *lin-42(ok2385)* allele, which disrupts the expression of the LIN-42A isoform (containing the SYQ/LT domains only) and deletes portions of the LIN-42B isoform (containing both the PAS and SYQ/LT domains), precociously execute stage-specific gene expression, fail to maintain periodic molting cycles and overexpress *Pmir::GFP-pest* transcriptional reporters [39](Figure S2). We interpret the complex phenotypes of *lin-42(ok2385)* animals as a reduction of the two modular activities of LIN-42 domains. Specifically, a reduction of LIN-42 PAS domain expression alters transcriptional output and deletion of LIN-42 isoforms which contain the SYQ/LT domains results in defects in periodic transcription. Further studies will be needed to define a specific molecular role for LIN-42 isoforms in maintaining normal periodic transcription.

Recent reports suggest that a significant portion of the *C. elegans* transcriptome is dynamically expressed [79]–[81]. The combined interpretation of these studies suggests that the post-embryonic expression of 5–20% of mRNAs is synchronized with the molting cycles. The conservation of this process implies that these temporal gene expression patterns confer fitness to an organism and raise a number of interesting questions regarding the nature of developmental gene regulation [81]. Because many genes whose oscillatory expression patterns are coupled to the molting cycle control cell fate decisions and cell metabolism in a dosage-dependent manner, it is interesting to speculate how their temporal expression patterns, and levels, are coordinated with their targets. We suggest that *lin-42* plays a fundamental role in this process for a wide range of non-coding and protein-coding genes. Because many of the transcriptional targets of *lin-42* include miRNAs, each of which may regulate a vast array of genes, the impact on the dynamic nature of the *C. elegans* transcriptome during development may be immense.

## Materials and Methods

### Nematode maintenance and genetics

*C. elegans* strains were grown under standard conditions and mutagenized as previously described [82]. Positional cloning of each suppressor was performed using standard methods [50]. Transformation of animals and integration of extrachromosomal arrays were performed as previously described [83]. See Text S1 for details of transgenic animals used in this manuscript.

### Microscopy

Lineage analysis and scoring of adult alae phenotypes were performed by picking staged animals of the indicated genotypes and monitoring seam cells derived from the V lineage as previously described [22]. All images were taken with an Axio Scope.A1 microscope equipped with a monochrome camera (Diagnostic Instruments Inc) and SPOT imaging software (SPOT Imaging Solutions). GFP images of the hypodermal and seam cells were used for further quantification of *Pmir::GFP-pest* intensity. The average GFP intensity per area (arbitrary units) was quantified using ImageJ64. For each reporter, 20 individual animals were analyzed per developmental stage. For *Plin-4::GFP-pest*, 10 hypodermal cell nuclei per animal per stage, or a total of 200 nuclei per time point, were used to calculate the average GFP intensity. For *Plet-7::GFP-pest*, 5 seam cells per animal per stage, or a total of 100 cells per time point, were used to calculate the average GFP intensity.

### Northern blots and TaqMan assays

Total RNA was isolated from staged populations of worms, and northern blots were performed as previously described [27]. Multiplex microRNA TaqMan assays were performed according to the manufacturer's specifications (Life Technologies) and quantified using the ABI 7900HT Fast Real-Time PCR system (Applied Biosystems). For each biological replicate (3 total), the means and standard deviations of the raw Ct values were calculated and the representative heatmap demonstrating the fold change signal was created using R packages (www.r-project.org).

### Developmental and behavioral assays

For the characterization of behavioral and GFP/mCherry reporter expression, animals were prepared in one of two ways. For analysis of L1-stage expression, embryos were bleached and staged according to standard protocols and then plated on standard NGM media with OP50 [84]. At indicated times after the release from L1 synchronization, L1-staged animals were imaged with an Axio Scope.A1 microscope. For analysis of the molting cycle and GFP/mCherry-pest reporter expression, individual animals (non-motile, non-pharyngeal pumping) were picked to fresh NGM plates seeded with 20 µL of OP50. Time courses were initiated for each animal after each animal ecdysed. To determine the active and lethargic periods of animals at each stage, the pumping rates of individual animals were observed for 30 s of every hour. GFP/mCherry-pest expression was then monitored using a Zeiss SteREO Discovery V12 microscope with appropriate filters. To prevent photo-bleaching, each animal was exposed to <3 s of UV light.

### Chip-Seq methods

See Text S1 for details.

## Supporting Information

Figure S1 Reduction of *lin-42* activity does not alter the levels of the *C. elegans* microRNA-specific Argonautes, ALG-1 and ALG-2. Parental animals (MJS13: *alg-1(gk214) In[alg-1p::rfp::alg-1::alg-1 3′UTR; alg-2p::gfp::alg-2::alg-2 3′UTR; pRF4]*) were fed bacteria expressing the indicated dsRNA and young adult F1 progeny were photographed with a CCD camera. (A) Representative images of both reporters in each RNAi experiment. (B) Quantitation of the average fluorescence for each reporter in the various RNAi experiments (n = 20 for each RNAi experiment).
(TIF)

Figure S2 *lin-42* mutants lead to the elevated expression of the *Plin-4::GFP-pest* reporter. Representative images of *Plin-4::GFP-pest* reporter expression in wild-type, *lin-42(n1089)* and *lin-42(ok2385)* animals. Each image was photographed with identical exposure times.
(TIF)

Figure S3 *Plet-7::GFP-pest* reporter expression is highly dynamic. *Plet-7::GFP-pest* expression begins at ∼14–15 hours, peaks by 19 hrs (near the end of the L1 stage) and ends after ∼21 hours. The peak of GFP-pest expression precedes the expression of the *Pcol-12::mCherry-pest* reporter. By 31 hours post-L1 arrest, the *Plet-7::GFP-pest* reporter is induced again.
(TIF)

Figure S4 Vulval morphologies used to stage animals in this manuscript. (A–H) Representative images of stage-specific vulval morphology used to classify animals in the transcriptional reporter activity assays. White triangles represent p-cells. White asterisk represents the anchor cell. Red triangle represents the initial invagination observed in L3 molting animals.
(TIF)

Table S1 Alleles of *lin-42*, origin and predicted alterations of *lin-42* isoforms.
(XLSX)

Table S2 List of high confidence LIN-42 ChIP peaks.
(XLSX)

Table S3 Annotation of GO terms for predicted LIN-42 ChIP-associated genes.
(XLSX)

Text S1 Includes details of transgenic animal construction, ChIP-Seq and data analysis for ChIP-Seq data.
(DOCX)
