# Supplementary figures and images for: LIN-42, the Caenorhabditis elegans PERIOD homolog, Negatively Regulates MicroRNA Transcription
Source: PLoS Genet. 2014 Jul 17;10(7):e1004486. doi: 10.1371/journal.pgen.1004486 (PMC4102445; doi:10.1371/journal.pgen.1004486)

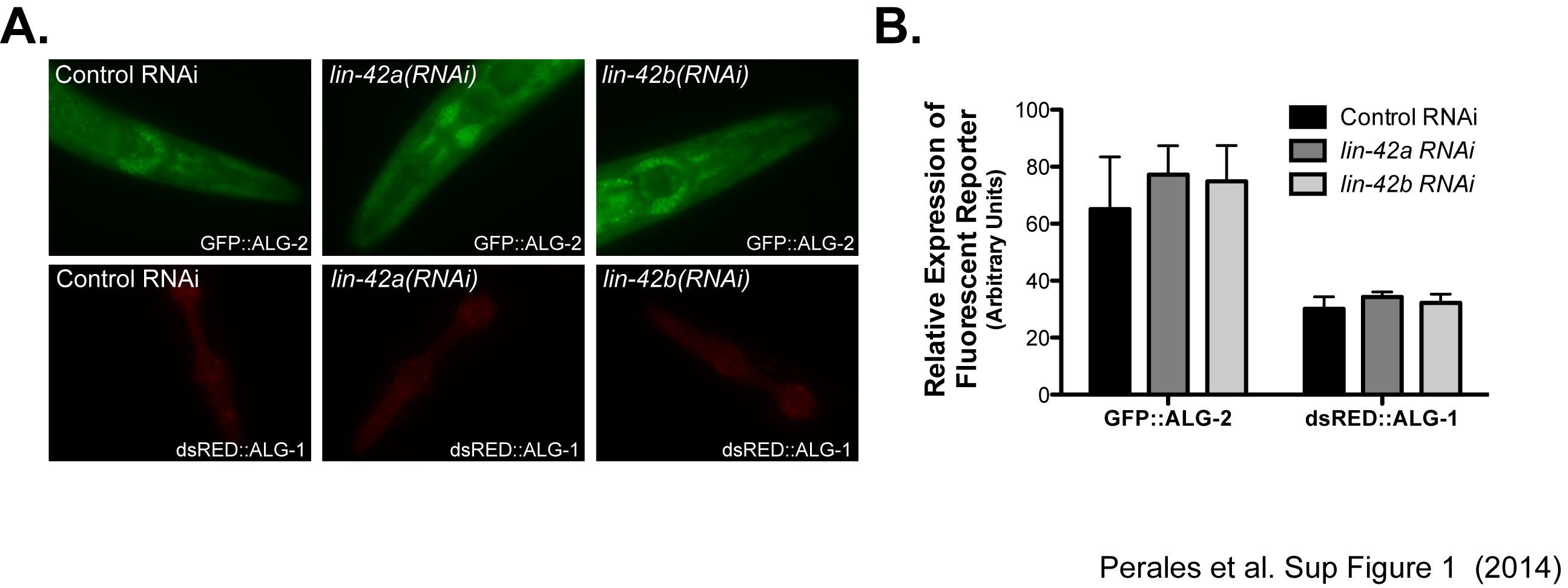

Supplement: Figure S1 — Reduction of lin-42 activity does not alter the levels of the C. elegans microRNA-specific Argonautes, ALG-1 and ALG-2. Parental animals (MJS13: alg-1(gk214) In[alg-1p::rfp::alg-1::alg-1 3′UTR; alg-2p::gfp::alg-2::alg-2 3′UTR; pRF4]) were fed bacteria expressing the indicated dsRNA and young adult F1 progeny were photographed with a CCD camera. (A) Representative images of both reporters in each RNAi experiment. (B) Quantitation of the average fluorescence for each reporter in the various RNAi experiments (n = 20 for each RNAi experiment). (TIF) [file pgen.1004486.s001.tif]

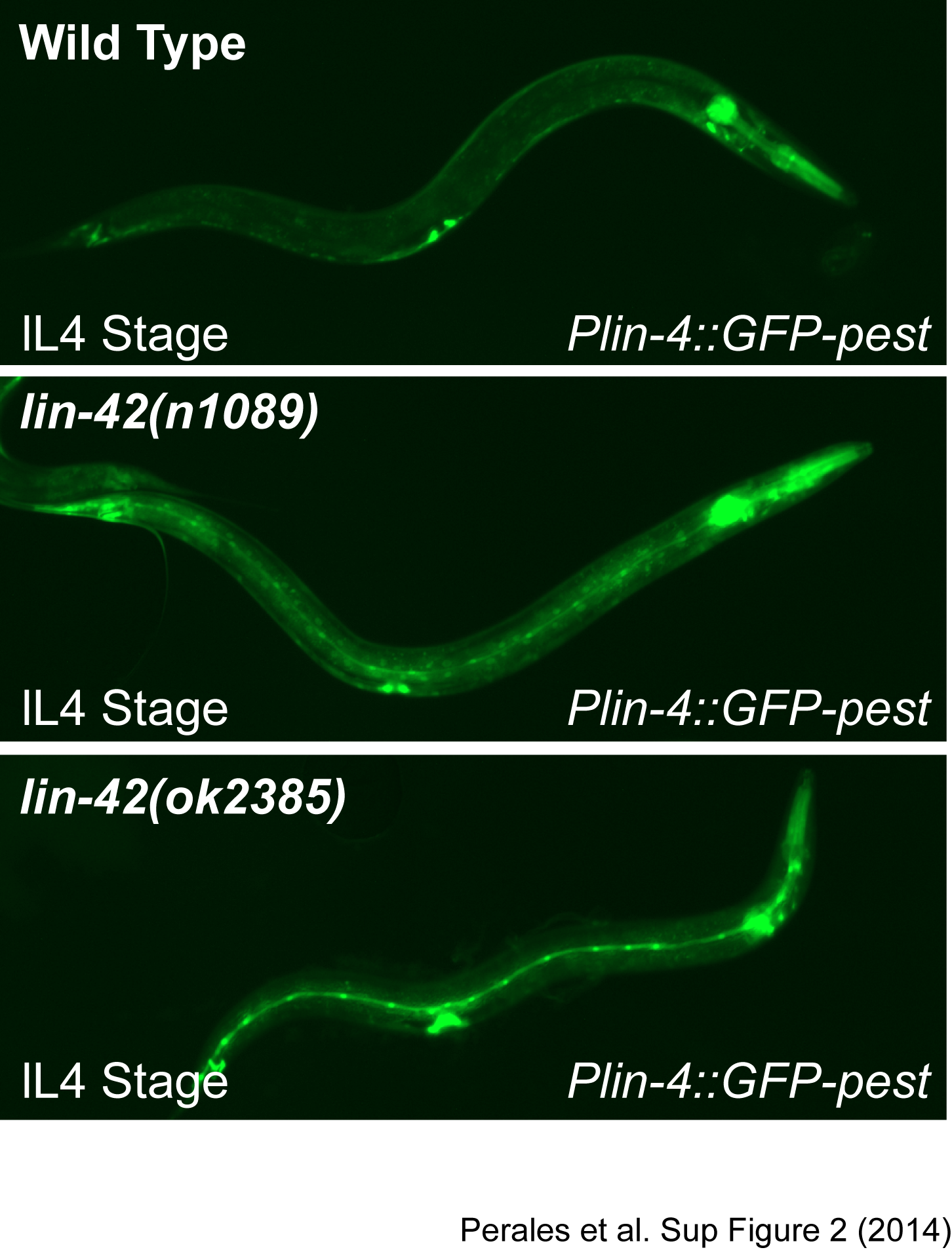

Supplement: Figure S2 — lin-42 mutants lead to the elevated expression of the Plin-4::GFP-pest reporter. Representative images of Plin-4::GFP-pest reporter expression in wild-type, lin-42(n1089) and lin-42(ok2385) animals. Each image was photographed with identical exposure times. (TIF) [file pgen.1004486.s002.tif]

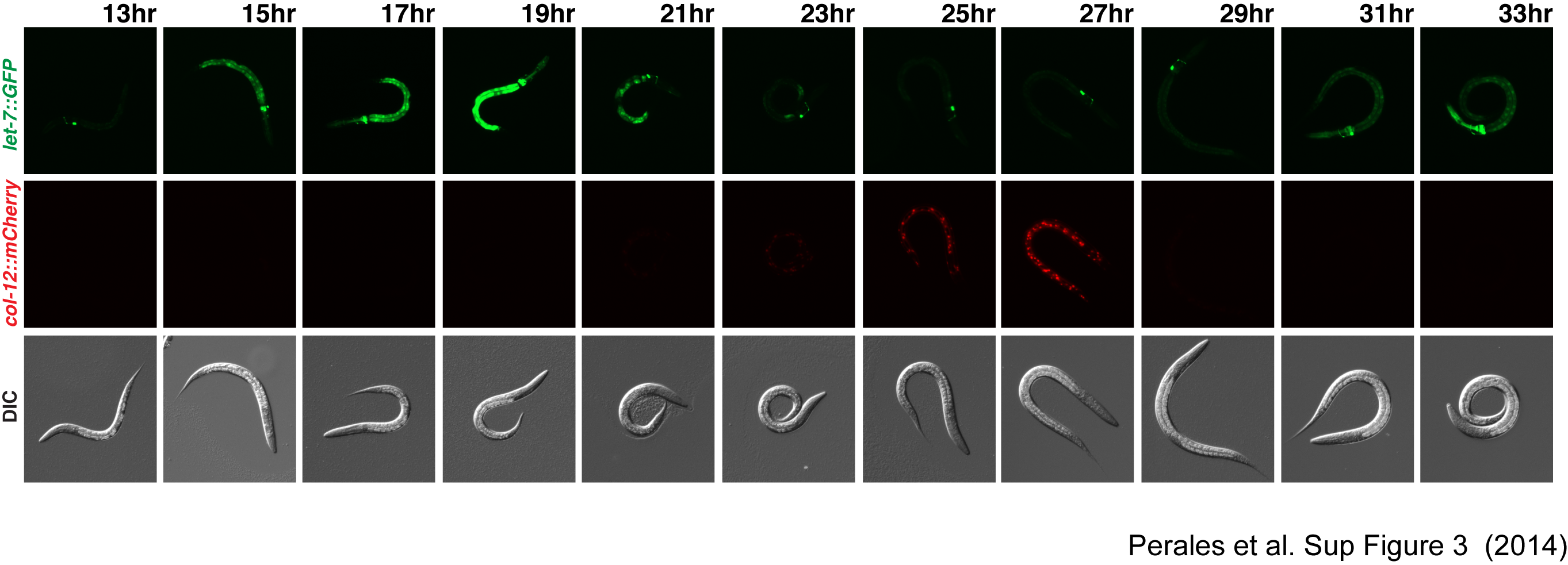

Supplement: Figure S3 — Plet-7::GFP-pest reporter expression is highly dynamic. Plet-7::GFP-pest expression begins at ∼14–15 hours, peaks by 19 hrs (near the end of the L1 stage) and ends after ∼21 hours. The peak of GFP-pest expression precedes the expression of the Pcol-12::mCherry-pest reporter. By 31 hours post-L1 arrest, the Plet-7::GFP-pest reporter is induced again. (TIF) [file pgen.1004486.s003.tif]

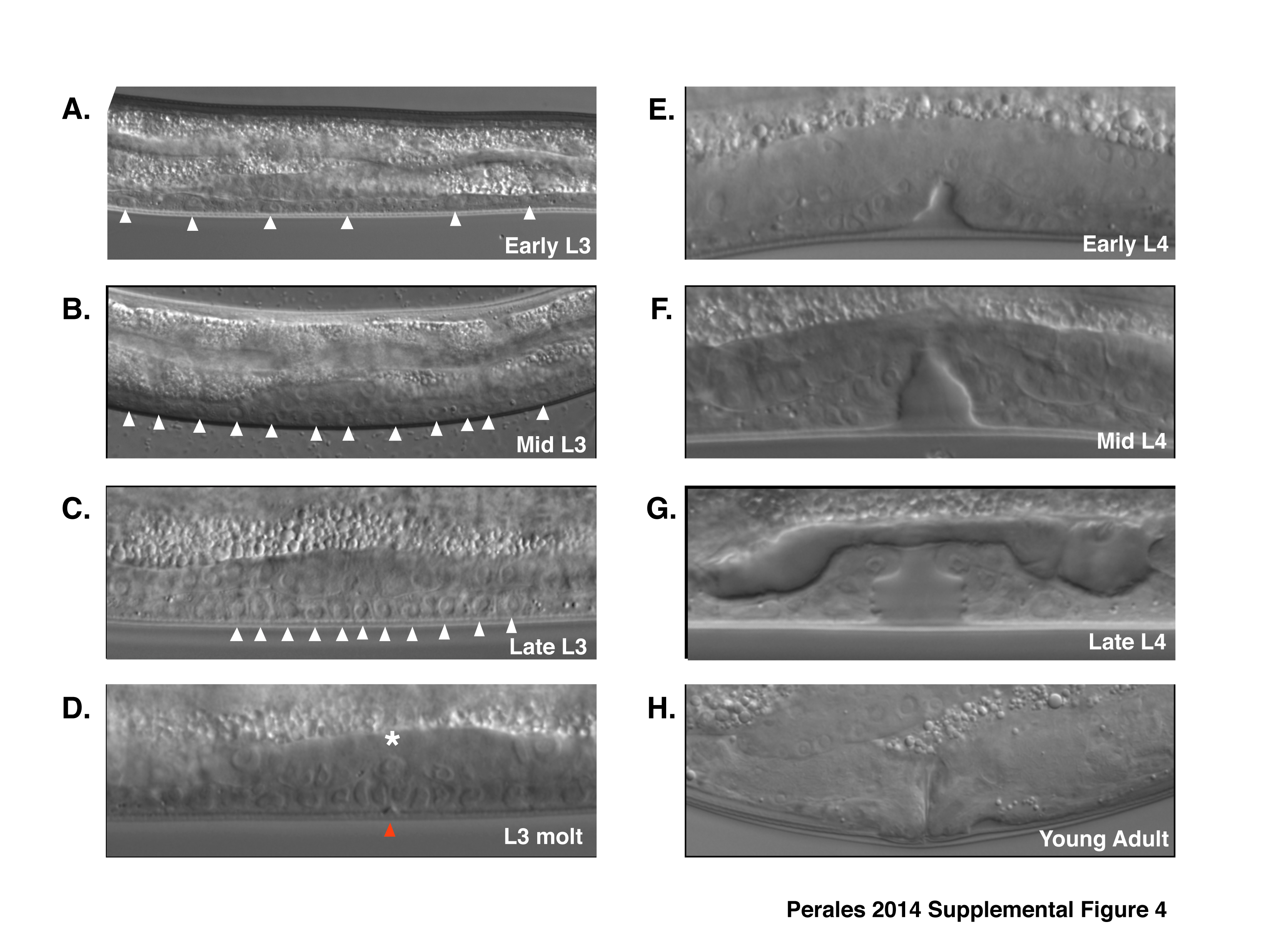

Supplement: Figure S4 — Vulval morphologies used to stage animals in this manuscript. (A–H) Representative images of stage-specific vulval morphology used to classify animals in the transcriptional reporter activity assays. White triangles represent p-cells. White asterisk represents the anchor cell. Red triangle represents the initial invagination observed in L3 molting animals. (TIF) [file pgen.1004486.s004.tif]
