# Supplementary material for: LIN-42, the Caenorhabditis elegans PERIOD homolog, Negatively Regulates MicroRNA Transcription
Source: PLoS Genet. 2014 Jul 17;10(7):e1004486. doi: 10.1371/journal.pgen.1004486 (PMC4102445; doi:10.1371/journal.pgen.1004486)
Supplement: Text S1 — Includes details of transgenic animal construction, ChIP-Seq and data analysis for ChIP-Seq data. (DOCX) [file pgen.1004486.s008.docx]

**Supplemental Information for:**

**LIN-42, the *Caenorhabditis elegans* PERIOD homolog, negatively regulates microRNA biogenesis**

**Roberto Perales^1^, Dana M. King^1,2^, Cristina Aguirre-Chen, C. M. Hammell^3^**

Cold Spring Harbor Laboratory, One Bungtown Road, Cold Spring Harbor, NY 11724

**Construction of transcriptional reporters:** The PEST domain was amplified from pAF207 (a generous gift from Dr. A. Frand, UCLA) and was used to create *NLS-GFP-pest::unc-54-3’UTR* (pCMH1225) and *NLS-mCherry-pest::unc-54-3’UTR* (pCMH1183). To make *Pcol-12::mCherry-pest* (pCMH1195), a 2kb genomic fragment containing the *col-12* promoter (chr V:10,422,005-10,429,220) was subcloned into the XmaI sites of pCMH1183. To make *Plin-4::GFP-pest* (pCMH1202), *Plet-7::GFP-pest* (pCMH1201), and *Pmir-1::GFP-pest* (pCMH1229), individual genomic fragments were subcloned into pCMH1225 as follows: for pCMH1202, a 1.8kb fragment (chr II:5,897,452..5,905,268) using XmaI sites; for pCMH1201, a 2.4kb fragment (chr X:14,742,029..14,749,245) using SalI sites; and for pCMH1229, a 2.6kb fragment (chr I:6,170,232..6,178,361) using XmaI sites. To make the *lin-42* transcriptional reporters, a 3.0kB BamHI fragment containing the *lin-42a* promoter sequence was subcloned into pCMH1125 to create pCMH1207 (*Plin-42a::GFP-pest*) and a 4.2kB BamHI fragment containing the *lin-42b/c* promoter was subcloned into pCMH1183 to create pCMH1210 (*Plin-42b/c::mCherry-pest*).

For each miRNA transcriptional reporter, DP38 *(unc-119(ed3))* animals were transformed with a mixture of DNA that included pIF9 (*unc-119(+)),* *ttx-3::GFP*, pCMH1195, and one of the *Pmir::GFP-pest* reporters. Strains containing integrated and extrachromosomal arrays of these transgenes are as follows: HML168 (*unc-119(ed3); cshIs11* *[Plin-4::GFP-pest; Pcol-12::mCherry-pest, ttx-3::GFP; unc-119(+)])*, HML181 (*lin-42(n1089); unc-119(ed3); cshIs11 [Plin-4::GFP-pest; Pcol-12::mCherry-pest, ttx-3::GFP; unc-119(+)])*, HML254 (*unc-119(ed3); cshIs13* *[Plet-7::GFP-pest; Pcol-12::mCherry-pest, ttx-3::GFP; unc-119(+)])*, HML255 (*lin-42(n1089); unc-119(ed3); cshIs13* *[Plet-7::GFP-pest; Pcol-12::mCherry-pest, ttx-3::GFP; unc-119(+)])*, HML176 (*unc-119(ed3); cshEx28 [Pmir-1::GFP-pest; Pcol-12::mCherry-pest, ttx-3::GFP; unc-119(+)]),* HML146 (*unc-119(ed3); cshEx21 [Plin-42a::GFP-pest; Plin-42b/c::mCherry-pest, ttx-3::GFP; unc-119(+)])*

**Antibody production:** Rabbit polyclonal antibodies were produced by immunizing rabbits with a peptide (CLENVHRLLKSQSRP) conjugated to KLH. This peptide sequence is found in the predicted amino acid sequences of LIN-42A and LIN-42B. Antibodies were affinity purified using SulfoLink Resin (Thermo Scientific, product #44995).

**ChIP-Seq Methods:** Hypochlorite treatment followed by overnight starvation was used to synchronize animals at L1. After two rounds of synchronization, L1 larvae were placed on 30, 100mm plates seeded with OP50 and allowed to grow until the late-L4 stage. 4 to 5mL of late-L4 animals were then collected and subsequently frozen in liquid nitrogen, pulverized with a BioPulverizer (BioSpec Products, catalog #59014H), and ground using a pre-chilled cryo-cup grinder (BioSpec Products, catalog #206) and a stainless steel ball (BioSpec Products, catalog #206SS138). The worm powder was then resuspended and crosslinked with 1.1% formaldehyde for 10 min at room temperature. The reaction was then quenched with 2.5M glycine, incubated at room temperature for 5min, and centrifuged at 4000rpms for 5min at 4ºC. The pellet was resuspended in 2mL of fresh, cold FA lysis buffer (50 mM HEPES/KOH pH 7.5, 1 mM EDTA, 1% Triton X-100, 0.1 % sodium deoxycholate; 150 mM NaCl) supplemented with protease inhibitors (Roche, catalog #04693132001). The extract was then sonicated with a Bioruptor® (Diagenonde, Model #ucd-200), aliquoted into pre-chilled 1.5mL tubes, and centrifuged at 13,000g for 15min at 4ºC. Extracts were either stored at -80ºC or used right away for chromatin immunoprecipitations. Protein concentrations of all extracts were measured using the Qbit® 2.0 fluorometer and the Qbit® protein assay kit (Invitrogen, catalog #Q32866 and #Q33211).

Chromatin immunoprecipitions of GFP-tagged LIN-42 were performed using either an anti-GFP antibody (Abcam, product #ab290) or a custom rabbit polyclonal antibody raised against LIN-42. An aliquot of 50uL of pre-cleared extract was saved for input DNA purification prior to immunoprecipitations. Pre-cleared extracts with ~2mg of total protein were supplemented with 0.1% SDS and 0.3% Sarkosyl, then incubated with antibodies and protein A/G agarose beads (Pierce, product #20423) overnight at 4ºC while rotating. Beads were collected by centrifugation at 3000rpm for 30sec, then washed twice with FA lysis buffer, once with FA lysis buffer with high salt (500 mM NaCl), once with TEL buffer (0.25 M LiCl, 1% NP-40, 1% sodium deoxycholate, 1 mM EDTA, 10 mM Tris-HCl, pH 8.0), and once with TE buffer (1% NP-40, 1% sodium deoxycholate, 1 mM EDTA, 10 mM Tris-HCl, pH 8.0). All washes were performed for 10min while rotating at room temperature in 1.4mL of the indicated buffer. Beads were subsequently resuspended in 100uL of TE buffer and treated with 1uL of RNAse A Solution (Qiagen, catalog #1042603) for 30min at 37ºC. After incubation, 1mL of TE buffer was added and beads were collected by centrifugation at 3000rpm for 30sec. To elute the immunocomplexes, beads were resuspended in 500uL of elution buffer (1% SDS, 250 mM NaCl, 10 mM Tris pH 8.0, 1 mM EDTA) and incubated at 65ºC in a water bath for 5min, then at room temperature for 10min while rotating. The beads were spun down by centrifugation at 3000rpm for 30sec and the supernatant was collected and transferred to a new pre-labeled 1.5mL tube. The protein/DNA complexes were reverse crosslinked by incubation overnight at 65ºC. Samples were then treated with 20ug of Puregene® Proteinase K (Qiagen, catalog #1042600) for 1hr at 37ºC. The immunoprecipitated DNA and input DNA were purified using the Wizard® SV Gel and PCR Clean-Up System (Promega, catalog #A9282) and resuspended in 50uL of molecular grade RNAse/DNAse free water.

ChIP-Seq libraries were prepared using the TruSeq® ChIP Sample Preparation Kit (Illumina, part #15034288). Quantification of the libraries was performed using the Qbit® 2.0 fluorometer with the dsDNA HS assay kit (Invitrogen, catalog #Q32851), and library quality was analyzed using the 2100 Bioanalyzer (Agilent Technologies, part #G2940CA). The resulting libraries were sequenced to produce reads of 76 nucleotides from a single end (SE) using the Illumina MiSeq platform. A table summarizing all the ChIP-Seq libraries produced for this study is in Supplemental Information Table 2.

Raw reads from the ChIP-Seq and input control sequences were aligned to the ce10 version of the *C. elegans* genome using the bowtie2 algorithm and parameters that were optimized to detect reads with approximately 3 mismatches per read and no more than 10 alignments genome-wide (-k10 -N 1).  Using these parameters, greater than 80% of the reads mapped for all samples.  Uniquely aligned reads were selected for input to the MACS peak calling algorithm, version 1.4, which automatically removes any duplicate sequences before calling ChIP peaks.  All peaks were called with respect to the input control samples, using default parameter settings.  Analysis of ChIP-Seq data revealed that (1) 946 peaks passed P-value and enrichment thresholds for LIN-42::GFP IP replicate 1, (2) 2580 peaks passed thresholds for LIN-42::GFP IP replicate 2, and (3) 1128 peaks passed thresholds for LIN-42-endogenous IP replicate 1. For the replicate GFP-IP samples, 413 peaks were detected in both samples.  Of these high confidence peaks, 323 were also detected in the endogenous LIN-42 IP samples, suggesting this list forms a short but high confidence list of LIN-42 target genes. Custom scripts were used to annotate the high confidence list of 413 peaks against the RefSeq list of gene models for ce10 (downloaded from the UCSC genome database in April of 2013), which was supplemented with the positions of all worm microRNA genes obtained from the Wormbase genome database ce10 in April of 2013.
